# Dysfunctional telomeres induce p53‐dependent and independent apoptosis to compromise cellular proliferation and inhibit tumor formation

**DOI:** 10.1111/acel.12476

**Published:** 2016-04-26

**Authors:** Yang Wang, Xinwei Wang, Elsa R. Flores, Jian Yu, Sandy Chang

**Affiliations:** ^1^ Department of Laboratory MedicineYale University School of MedicineNew HavenCTUSA; ^2^ University of Pittsburgh School of MedicineUniversity of Pittsburgh Cancer InstituteHillman Cancer Center Research PavilionPittsburghPAUSA; ^3^ Department of Molecular & Cellular OncologyDepartment of Translational Molecular PathologyGraduate School of Biomedical SciencesU.T. MD Anderson Cancer CenterHoustonTXUSA; ^4^ Departments of Pathology and Molecular Biophysics and BiochemistryYale University School of MedicineNew HavenCTUSA

**Keywords:** apoptosis, cellular senescence, DNA damage, molecular biology of aging, stem cells, telomeres

## Abstract

Aging is associated with progressive telomere shortening, resulting in the formation of dysfunctional telomeres that compromise tissue proliferation. However, dysfunctional telomeres can limit tumorigenesis by activating p53‐dependent cellular senescence and apoptosis. While activation of both senescence and apoptosis is required for repress tumor formation, it is not clear which pathway is the major tumor suppressive pathway *in vivo*. In this study, we generated *E*μ*‐myc; Pot1b* ^***∆/∆***^ mouse to directly compare tumor formation under conditions in which either p53‐dependent apoptosis or senescence is activated by telomeres devoid of the shelterin component Pot1b. We found that activation of p53‐dependent apoptosis plays a more critical role in suppressing lymphoma formation than p53‐dependent senescence. In addition, we found that telomeres in *Pot1b* ^*∆/∆*^ *; p53* ^*−/−*^ mice activate an ATR‐Chk1‐dependent DNA damage response to initiate a robust p53‐independent, p73‐dependent apoptotic pathway that limited stem cell proliferation but suppressed B‐cell lymphomagenesis. Our results demonstrate that in mouse models, both p53‐dependent and p53‐independent apoptosis are important to suppressing tumor formation.

## Introduction

Aging is due in part to increased accumulation of damaged DNA in highly proliferative tissues, leading to compromised tissue homeostasis, disease, and frailty (Kirkwood, 2008). One source of endogenous DNA damage comes from telomeres, protein‐TTAGGG repetitive DNA complexes that cap the ends of eukaryotic chromosomes (Greider, 1996). Telomeres play important roles in maintaining genome stability by preventing the activation of DNA damage checkpoints that induce p53‐dependent cell cycle arrest and apoptosis (Chin *et al*., 1999; Artandi *et al*., 2000; Gonzalez‐Suarez *et al*., 2000). They are synthesized by the enzyme telomerase, and interact with a number of telomere‐specific binding proteins that form a complex, termed shelterin, which protects telomeres from inappropriately activating DNA damage responses (Deng *et al*., 2008; Palm & de Lange, 2008; Martinez & Blasco, 2010). Shelterin components that interact with double‐stranded telomeric DNA include TRF1 and TRF2‐RAP1, while the single‐stranded (ss) telomere DNA‐binding protein POT1 forms a heterodimer with TPP1. TIN2 serves to bridge the POT1‐TPP1 complex with TRF1 and TRF2 and stabilizes POT1‐TPP1 to promote genome stability (Baumann & Cech, 2001; O'Sullivan & Karlseder, 2010).

POT1 homologs are highly conserved evolutionarily and have been identified in almost all eukaryotes. The mouse possesses two POT1 proteins, Pot1a and Pot1b while humans possess a single POT1 protein (He *et al*., 2006; Hockemeyer *et al*., 2006; Wu *et al*., 2006). All POT1 proteins bind to the 3′ ss TTAGGG repeats that mark the ends of mammalian telomeres with high affinity (Loayza *et al*., 2004; He *et al*., 2006; Wu *et al*., 2006; Palm *et al*., 2009; Nandakumar *et al*., 2010). In addition, both human POT1 and mouse Pot1b modulates the 5′ nucleolytic processing of the telomeric C‐strand and consequently regulates the formation of the 3′ ss overhang (He *et al*., 2006; Hockemeyer *et al*., 2008; Palm *et al*., 2009). The ability of POT1 to interact and limit the generation of the 3′ overhang is essential to prevent the activation of the ataxia telangiectasia mutated and Rad3‐related protein (ATR)‐kinase‐dependent DNA damage signaling at telomeres. Removal of POT1 results in the recruitment of replication protein A (RPA), the sensor of the ATR pathway, to telomeres. It also promotes the activation of downstream signaling kinases, including Chk1, that then signal additional effector proteins that these dysfunctional telomeres now resemble double‐stranded DNA breaks (Wu *et al*., 2006; Denchi & de Lange, 2007; Guo *et al*., 2007; Flynn *et al*., 2011; Thanasoula *et al*., 2012). In mouse models, dysfunctional telomeres due to Pot1b loss activate p53‐dependent DNA damage checkpoint responses that compromise stem cell proliferation, leading to premature aging phenotypes and compromised organismal lifespan (Wang *et al*., 2011, 2013). These results reveal an essential role for POT1 in preventing the inappropriate activation of the ATR‐mediated, p53‐dependent DNA damage response at telomeres of highly proliferative cells.

The POT1‐TPP1 complex also prevents inappropriate repair of dysfunctional telomeres, manifested as chromosome fusions in metaphase spreads. Both activation of the ATR kinase, and the generation of 3′ overhangs, are essential to promote alternative, nonhomologous end joining (A‐NHEJ)‐mediated repair of mouse telomeres devoid of *Pot1a/b* (Rai *et al*., 2010, 2011; Sfeir & de Lange, 2012). A‐NHEJ is a microhomology based error‐prone repair pathway that promotes gross chromosomal abnormalities, including the generation of large deletions, insertions, and translocations, resulting in elevated genome instability (Bennardo *et al*., 2008; Simsek & Jasin, 2010; Symington & Gautier, 2011). Importantly, point mutations in human POT1 predicted to inactivate its telomere protective functions have been found in both Chronic Lymphocytic Lymphoma and familial melanoma (Quesada *et al*., 2012; Ramsay *et al*., 2013; Robles‐Espinoza *et al*., 2014; Shi *et al*., 2014). Coupled with observations that deletion of Pot1a or Pot1b promotes end‐to‐end chromosome fusions that is tumor promoting in p53 mutant tumor prone mouse models (Wu *et al*., 2006; Hockemeyer *et al*., 2008; He *et al*., 2009; Wang *et al*., 2011), these results highlight the importance of POT1 in promoting genome stability to prevent the onset of cancer.

The p53 tumor suppressor is functionally inactivated in the majority of human cancers. Double strand breaks including those generated by the expression of oncogenes initiate p53‐dependent cellular checkpoint responses to induce a tumorigenic barrier (Halazonetis *et al*., 2008). Consequently, mice with critically shortened telomeres are resistant to tumor development due to the activation of p53‐dependent cellular senescence and apoptosis programs. Activation of p53‐dependent downstream targets, including the cyclin‐dependent kinase inhibitor CDKN1A/p21^WAF1/CIP1^ (p21) by dysfunctional telomeres, results in G1 cell cycle arrest and the onset of cellular senescence to potently suppresses tumor initiation and progression *in vivo* (Cosme‐Blanco *et al*., 2007; Feldser & Greider, 2007). In addition, dysfunctional telomeres also activate p53‐dependent apoptotic proteins, including BAX and PUMA, to limit stem cell proliferation and suppress tumorigenesis (Chin *et al*., 1999; Gonzalez‐Suarez *et al*., 2000; Rudolph *et al*., 2001; Begus‐Nahrmann *et al*., 2009; Sperka *et al*., 2012a). While dysfunctional telomere‐induced p53‐dependent apoptosis and senescence are both required for repress tumor formation, it is not known which pathway is the dominant tumor suppressive pathway *in vivo*.

In this study, we generated *E*μ*‐myc; Pot1b* ^*∆/∆*^ mouse to directly compare tumor formation under conditions in which either p53‐dependent apoptosis or cellular senescence is activated by dysfunctional telomeres. Overexpression of the Myc oncogene in B cells results in high penetrance formation of B‐cell lymphomas within 5 months, thus providing an excellent model to test mechanisms that limit tumor growth (Harris *et al*., 1988). We found that p53‐dependent apoptosis plays a more important role in lymphoma suppression than p53‐dependent senescence. In addition, we also discovered that dysfunctional telomeres in *Pot1b* ^*∆/∆*^ *; p53* ^*−/−*^ mice can activate a robust p53‐independent, the p53 ortholog p73‐dependent apoptotic program to negatively impact upon tissue proliferative capacity but is able to repress B‐cell lymphomagenesis. In addition to uncovering the importance of dysfunctional telomere‐induced apoptosis in tumor suppression, our results also reveal a previously unappreciated role for p73 in mediating p53‐independent suppression of B‐cell lymphomas.

## Results

### Near complete suppression of lymphomagenesis in *E*μ*‐myc; Pot1b* ^*∆/∆*^ mice

**Figure 1:**
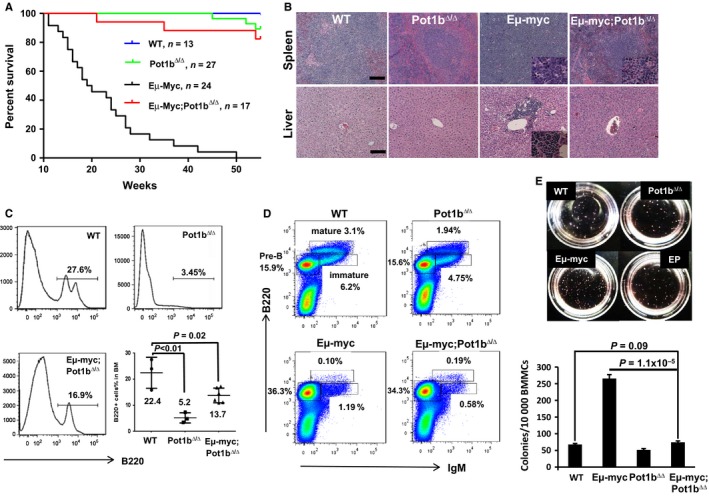
Inhibition of tumorigenesis in *Eμ‐myc; Pot1b* ^*Δ/Δ*^ mice promotes lifespan extension. (A) Kaplan–Meier analysis of tumor free survival of WT,* Pot1b* ^*Δ/Δ*^, *Eμ‐myc* and *Eμ‐myc; Pot1b* ^*Δ/Δ*^ mice. All mouse cohorts were monitored for at least 55 weeks and sacrificed when moribund. A significant increase in lifespan of *Eμ‐myc; Pot1b* ^*Δ/Δ*^ over *Eμ‐myc* mice was observed (*P* < 0.0001). The log‐rank test was used to calculate statistical significance. (B) Hematoxylin and eosin (H&E) staining of tissues from *Eμ‐myc* mice demonstrates malignant B‐cell infiltration of the spleen and metastasis to the liver. Mice of other genotypes show normal spleen and liver architectures (magnification × 10). Scale bar: 25 μm. Insets show magnified (40 ×) malignant cells. (C) First three panels: representative FACS analysis of B220 positive cells in whole bone marrow (WBM) of 55 weeks old mice of the indicated genotypes. Significantly reduced number of B220+ cells were found in the *Eμ‐myc Pot1b* ^*Δ/Δ*^ mice compared with those of WT (*P* = 0.02). Last panel: summary of results. A two‐tailed Student's *t* test was used to calculate statistical significance. WT:* n* = 3. Pot1b^*Δ/Δ*^: *n* = 4. Eμ‐myc Pot1b^*Δ/Δ*^: *n* = 6. (D) Representative FACS analysis of pre‐B (B220+, IgM−), immature B (B220 lower, IgM+) and mature B cells (B220 higher, IgM+) in WBM from mice of the indicated genotypes. A summary of the data is shown in Table 1. Each experiment was repeated at least three times. (E) (Top) Representative images of colony forming assays after 12 days in culture in M3630 media specific for pre‐B cells growth. Cell genotypes are indicated. EP:* Eμ‐myc; Pot1b* ^*Δ/Δ*^. (Bottom) Quantification of the number of colonies. No significant differences exist between WT and *Eμ‐myc; Pot1b* ^*Δ/Δ*^ cells (*P* = 0.09). There is a significant difference between *Eμ‐myc* and *Eμ‐myc; Pot1b* ^*Δ/Δ*^ cells (*P* = 1.1 × 10^−5^). Results are mean from three experiments, and error bars represent SEM. A two‐tailed Student's *t* test was used to calculate statistical significance.

We crossed *Pot1b* ^*∆/∆*^ mice with *E*μ*‐myc* transgenic mice and monitored cohorts of *Pot1b* ^*∆/∆*^ *, E*μ*‐myc* and *E*μ*‐myc; Pot1b* ^*∆/∆*^ mice for lymphoma development. While B‐cell lymphomas were never observed in *Pot1b* ^*∆/∆*^ mice, *E*μ*‐myc* mice rapidly succumbed to fulminant B‐cell lymphoma with a median onset 19.5 weeks. In sharp contrast, 14 of 17 *E*μ*‐myc; Pot1b* ^*∆/∆*^ mice remained tumor free by 55 weeks of age (*P* < 0.0001, Fig. 1A). H&E staining revealed that while malignant B cells from *E*μ*‐myc* mice completely infiltrated the spleens and livers, lymphoma infiltrate was undetectable in *E*μ*‐myc; Pot1b* ^*∆/∆*^ mice (Fig. 1B). To understand why deletion of *Pot1b* potently suppressed Myc‐induced lymphomagenesis, we used the B‐cell marker B220 to monitor the number of B cells in the bone marrows (BM) of similarly aged mouse cohorts. Compared to wild‐type (WT) mice, tumor‐free 50‐week‐old *E*μ*‐myc; Pot1b* ^*∆/∆*^ mice displayed significant decrease in the percentage of B cells (22.4% ± 3.4% vs. 13.7% ± 1.1%, *P* = 0.02) (Fig. 1C). We postulated that this diminished B‐cell count could be due to either a block in *E*μ*‐myc; Pot1b* ^*∆/∆*^ B‐cell differentiation or due to reduced cellular proliferation. To distinguish between these two possibilities, we first investigated the status of the B‐cell population in the BM of our mouse cohorts. Of the three stages of B cells (pre‐B, immature, and mature) that were evaluated, a significant expansion of the pre‐B‐cell compartment was detected in the BM from 6‐week‐old *E*μ*‐myc* mice (Fig. 1D and Table 1). Expansion of this compartment is characteristic of Myc‐induced B‐cell activation (Harris *et al*., 1988) and was not observed in either WT or *Pot1b* ^*∆/∆*^ mice. A similar expansion of the pre‐B‐cell compartment was also observed in *E*μ*‐myc; Pot1b* ^*∆/∆*^ mice, suggesting that malignant B‐cell clones, while present in *E*μ*‐myc; Pot1b* ^*∆/∆*^ mice, were incapable of forming lymphomas (Fig. 1D and Table 1). This hypothesis was further reinforced when we deleted a single allele of p53 in this mouse cohort. In this genetic setting, fulminant B‐cell lymphoma infiltrating distant organs was detected in 100% of *E*μ*‐myc; Pot1b* ^*∆/∆*^ *; p53* ^*+/−*^ mice, revealing that activation of p53‐dependent checkpoint by dysfunctional telomeres is essential to repress lymphomagenesis (Fig. S1A, Supporting information). We next asked whether pre‐B cells in the bone marrow of our mice were able to form colonies *in vitro*, a robust measure of cellular proliferative capacity. Compared to the number of colonies formed by *E*μ*‐myc* B cells, *E*μ*‐myc; Pot1b* ^*∆/∆*^ B cells formed significantly fewer number of colonies (265 ± 11 vs. 74 ± 4, *P* = 1.1 × 10^*−*5^) (Fig. 1E). In fact, the number of colonies formed by *E*μ*‐myc; Pot1b* ^*∆/∆*^ B cells was similar to those formed by WT and *Pot1b* ^*∆/∆*^ B cells (WT: 67.6 ± 3.2; *Pot1b* ^*∆/∆*^: 51.3 ± 4.0). These data suggest that the potent suppression of lymphomagenesis in *E*μ*‐myc; Pot1b* ^*∆/∆*^ mice was not due to diminished production of malignant pre‐B‐cell clones, but rather due to decreased proliferative capacity.

**Table 1:** B‐cell differentiation status in the bone marrow

Genotype	Pre‐B	Immature	Mature	*P* value
WT	17.2% ± 1.1%	6.6% ± 0.81%	3.7% ± 0.67%	
Pot1b^∆/∆^	15.4% ± 0.82%	5.6% ± 1.51%	2.7% ± 0.59%	0.95^a^
Eμ‐myc	37.6% ± 3.1%	1.4% ± 0.45%	0.10% ± 0.07%	0.0023^a^
Eμ‐myc; Pot1b^∆/∆^	33.5% ± 4.0%	0.86% ± 0.26%	0.20% ± 0.06%	0.0012^a^; 0.99^b^

a Compared with WT.

b Compared with Eμ‐myc (by two‐way ANOVA).

### Elevated telomere damage and activation of p53‐dependent cellular checkpoints in *E*μ*‐myc; Pot1b* ^*∆/∆*^ B cells

**Figure 2:**
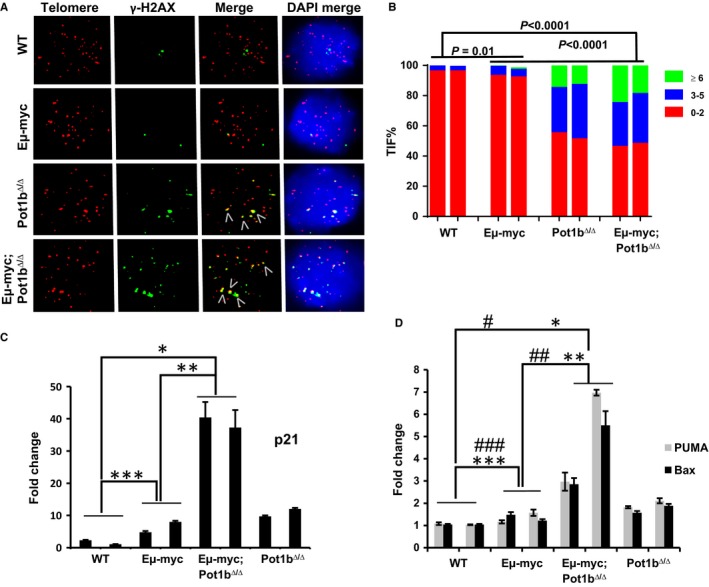
Enhanced DNA damage response in *Eμ‐myc; Pot1b* ^*Δ/Δ*^ B220+ cells. (A) Representative TIF images of B‐ cells from mice of the indicated genotypes. Cells were stained with anti‐ γ‐H2AX antibody (green), telomere PNA‐FISH probe [Tam‐OO‐(CCCTAA)_4_ (red)] and DAPI (blue). B‐cells were harvested from at least three 6–8 weeks old mice per genotype. (B) Quantification of percentage sorted B220+ cells displaying γ‐H2AX‐positive TIFs. A minimum of 150 nuclei were scored per genotype. A higher frequency of TIFs was detected in *Eμ‐myc; Pot1b* ^*Δ/Δ*^ B220+ cells compared with *Eμ‐myc* cells (*P* < 0.001). 2‐way ANOVA was performed to calculate statistical significance. (C) and (D) Real‐time PCR quantification of mRNA expression levels of p21 (C), PUMA and Bax (D) in sorted B220+ cells from 6 to 8 weeks old mice of the indicated genotypes. Each experiment was repeated in triplicate. Error bars represent SEM. 2‐way ANOVA was performed to calculate statistical significance. For (C), *P = 4.7 × 10^−8^, **P = 2.4 × 10^−7^ and ***P = 1.5 × 10^−4^. For (D), PUMA: ^#^ *P* = 1.5 × 10^−4^; ^##^ *P* = 2.7 × 10^−3^; ^###^ *P* = 1.4 × 10^−2^. BAX: *P = 6.7 × 10^−4^; **P = 1.4 × 10^−3^; ***P = 2.8 × 10^−3^.

To test the hypothesis that dysfunctional telomeres present in *E*μ*‐myc; Pot1b* ^*∆/∆*^ B cells activate a p53‐dependent DNA damage response (DDR) to suppress proliferation of precursor oncogenic B‐cell clones, we sorted B220 + cells from the BM and used the dysfunctional telomere‐induced DNA damage (TIF) assay (d'Adda di Fagagna *et al*., 2003; Takai *et al*., 2003) to determine the functional status of telomeres in 6‐week‐old mice. As expected, the DNA damage marker γ‐H2AX did not associate appreciably with telomeres in B cells isolated from WT or *E*μ*‐myc* mice (Fig. 2A). In contrast*, a* dramatic increase in the number of TIFs was observed in B cells from both *Pot1b* ^*∆/∆*^ and *E*μ*‐myc; Pot1b* ^*∆/∆*^ mice*. In total, 36% of Pot1b* ^*∆/∆*^ *B cells displayed 2–5 TIFs while 15% displayed more than 6 TIFs. In E*μ*‐myc; Pot1b* ^*∆/∆*^ B cells, 34% displayed 2–5 TIFs while 25% displayed more than 6 TIFs (*P* < 0.0001, Fig. 2B). As dysfunctional telomeres activate a p53‐dependent DDR, we next determined the expression of downstream targets of p53, including the cyclin‐dependent kinase inhibitor CDKN1A/p21^WAF1/CIP1^ (p21), BAX, and PUMA, in isolated B cells by real‐time RT–PCR. Compared to WT and *E*μ*‐myc* B cells, where only a slight increase in the expression of p21, PUMA, and BAX was observed, a dramatic increase in the expression of p21, PUMA, and BAX was evident in *E*μ*‐myc; Pot1b* ^*∆/∆*^ B cells (40.4‐fold increase for p21, 6.97‐fold increase for PUMA, and 5.5‐fold increase for BAX) (Figs 2C,D). Taken together, these results suggest that the chronic activation of both p53‐dependent cell cycle arrest/cellular senescence and apoptotic programs by dysfunctional telomeres lacking Pot1b may be responsible for the potent suppression of B‐cell lymphomagenesis observed in *E*μ*‐myc; Pot1b* ^*∆/∆*^ mice.

### Cellular proliferation is compromised in *Pot1b* ^*∆/∆*^ *; p53* ^*R172P*^ mice

**Figure 3:**
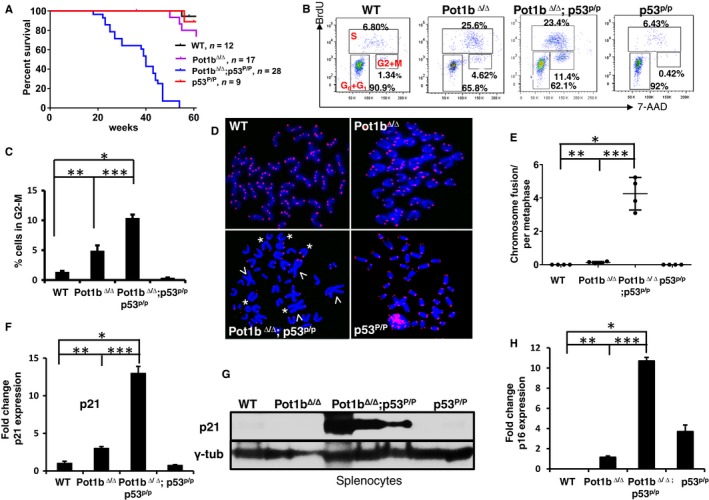
Deletion of Pot1b promotes cell cycle arrest and limits cellular proliferation. (A) Kaplan–Meier survival analysis of mice of the indicated genotypes. All mice were monitored for 60 weeks and sacrificed when moribund. A log‐rank test was used to calculate statistical significance. The lifespan of *Pot1b* ^*Δ/Δ*^ *; p53* ^*P/P*^ mice was significantly shorter than that of *p53* ^*P/P*^ mice (*P* < 0.0001). (B) BrdU cell‐cycle analysis of LSK cells isolated from 30 to 35 weeks old mice of the indicated genotypes. Representative FACS plots from experiments with at least three mice in each genotype are shown. Percentage of cells in various phases of the cell cycle are indicated. For example, *Pot1b* ^*Δ/Δ*^ *; p53* ^*P/P*^ mice have 11.4% BM LSK cells in the G2/M phase of the cell cycle. (C) Quantification of cells in (B) undergoing G2/M cell cycle arrest. A two‐tailed student's *t* test was used to calculate statistical significance. *P* values: *P = 1.08 × 10^−5^; **P = 2.4 × 10^−3^; ***P = 8.1 × 10^−4^. (D) Telomere PNA‐FISH of metaphase spreads showing end‐to‐end chromosome fusions (arrowheads) and telomere signal‐free ends (SFEs) (*). Only representative SFEs are indicated. At least four mice were used for each genotype, and a minimum of 40 metaphases scored per genotype. (E) Quantification of chromosome fusions in (D). A two‐tailed student's *t* test was used to calculate statistical significance. *P* values: *P = 2.0 × 10^−4^; **P = 1.6 × 10^−2^; ***P = 1.0 × 10^−4^. (F) Real‐time RT‐PCR analysis of p21 expression levels in sorted LSK cells from 30 to 35 weeks old indicated mice. Each experiment was repeated in triplicate. Error bars represent SEM. A two‐tailed student's *t* test was used to calculate statistical significance. *P = 4.5 × 10^−5^; **P = 4.8 × 10^−4^; ***P = 2.4 × 10^−5^. (G) Western blot for p21expression in splenocytes from mice of indicated genotypes. γ‐tubulin was used as the loading control. (H) Real‐time RT‐PCR analysis of p16 expression levels in sorted LSK cells from 30 to 35 weeks old indicated mice. Each experiment was repeated in triplicate. Error bars represent SEM. A two‐tailed student's *t* test was used to calculate statistical significance. *P = 1.6 × 10^−8^; **P = 1.1 × 10^−4^; ***P = 7.9 × 10^−7^.

To determine which antitumor program (apoptosis or cellular senescence) activated by dysfunctional telomeres is more important to suppress *E*μ*‐myc*‐induced lymphomagenesis, we first generated and characterized the Pot1b^***∆/∆***^; p53^R172P/R172P^ mice. The p53^R172P^ point mutation substitutes the wild‐type p53 alleles with mutant p53^R172P^, in which arginine at position 172 is replaced with a proline residue. p53^R172P^ (abbreviated as p53^P^) is the ortholog of a human p53 mutant that is completely defective for p53‐dependent apoptosis but still able to mediate cell cycle arrest/cellular senescence (Liu *et al*., 2004b; Post *et al*., 2010). Pot1b^***∆/∆***^; p53^P/P^ mice do not develop spontaneous tumors but instead die prematurely (median lifespan of ~40 weeks, *P* < 0.0001) (Fig. 3A). Preliminary characterizations of aging phenotypes revealed that these mice were born smaller than WT, *Pot1b* ^***∆/∆***^ or *p53* ^*P/P*^ littermate controls and display premature age‐related phenotypes, including diminished body size, kyphosis, and alopecia (Fig. S2A, Supporting information). Examination of total nucleated bone marrow (TBM) cells revealed that *Pot1b* ^*∆/∆*^ *; p53* ^*P/P*^ mice displayed significantly fewer proliferative TBM cells than those from WT littermates (6.92 ± 0.3 × 10^7^ cells for WT; 4.03 ± 0.59 × 10^7^ cells for *Pot1b* ^*∆/∆*^ *; p53* ^*P/P*^, *P* = 2.1 × 10^*−*5^) (Fig. S3A, Supporting information). To investigate whether this proliferative defect was due to loss of hematopoietic stem cell populations, we examined Lin^−^, Sca‐1^+^, c‐kit^+^ (LSK) cells, a population enriched in hematopoietic stem cells and multipotent progenitors. FACS analysis revealed that 25.6% of *Pot1b* ^*∆/∆*^ and 23.4% of *Pot1b* ^*∆/∆*^ *; p53* ^*P/P*^ LSK cells were in S‐phase due to increased cellular proliferation as a consequence of enhanced depletion of this cellular compartment from telomere dysfunction‐induced apoptosis (16). In total, 11.4% of *Pot1b* ^*∆/∆*^ *; p53* ^*P/P*^ LSK cells, compared to 4.6% of *Pot1b* ^*∆/∆*^ LSK cells, were found in the G2/M phase of the cell cycle (Figs 3B,C and S3B). To ascertain whether uncapped telomeres contributed to this phenotype, we examined metaphase chromosome spreads isolated from WT, *Pot1b* ^*∆/∆*^; *p53* ^*P/P*^ and *Pot1b* ^*∆/∆*^ *; p53* ^*P/P*^ TBM for signs of telomere dysfunction. Telomere PNA‐FISH analysis of age‐matched *Pot1b* ^*∆/∆*^ *; p53* ^*P/P*^ TBM metaphase spreads revealed a significant increase in the number of elevated end‐to‐end chromosome fusions and telomere signal‐free chromosome ends (Figs 3D,E and S3C). SA‐β‐galactosidase staining revealed significantly increased number of senescent *Pot1b* ^*∆/∆*^ *; p53* ^*P/P*^ BM cells (Fig. S2B,C). Western and RT–PCR analysis revealed significantly increased expression of p21 in *Pot1b* ^*∆/∆*^ *; p53* ^*P/P*^ LSK cells, splenocytes, and kidneys (Figs 3F,G and S2D). In addition, we observed increased p16 expression in *Pot1b* ^*∆/∆*^ *; p53* ^*P/P*^ LSK BM cells (Fig. 3H). Taken together, these results suggest that highly proliferative cells unable to activate p53‐dependent apoptosis respond to *Pot1b* deletion by promoting cellular senescence to compromise cellular proliferation.

### Complete suppression of lymphomagenesis in *E*μ*‐myc; Pot1b* ^∆/∆^ mice requires the activation of both p53‐dependent cell cycle arrest/cellular senescence and apoptosis

**Figure 4:**
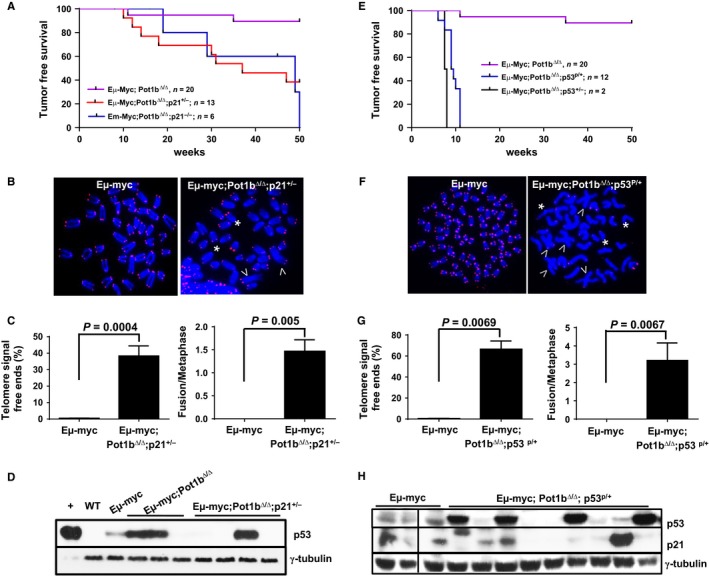
Both p53 dependent apoptosis and cellular senescence are required for complete tumor suppression in *Eμ‐myc; Pot1b* ^*Δ/Δ*^ mice. (A) Kaplan–Meier survival analysis of mice of the indicated genotypes, monitored over 50 weeks and sacrificed when moribund. Compared to the lifespan of *Eμ‐myc; Pot1b* ^*Δ/Δ*^ mice, the lifespan of *Eμ‐myc; Pot1b* ^*Δ/Δ*^ *; p21* ^*+/−*^ and *Eμ‐myc; Pot1b* ^*Δ/Δ*^ *; p21* ^*−/−*^ mice were significantly reduced (both *P* values < 0.01). The survival between *Eμ‐myc; Pot1b* ^*Δ/Δ*^ *; p21* ^*+/−*^ and *Eμ‐myc; Pot1b* ^*Δ/Δ*^ *; p21* ^*−/−*^ mice was not significantly different (*P* = 0.08). (B) Telomere FISH analysis of metaphase chromosome spreads from *Eμ‐myc* and *Eμ‐myc; Pot1b* ^*Δ/Δ*^ *; p21* ^*+/−*^ lymphomas using Tam‐OO‐(CCCTAA)_4_ (red) and DAPI (blue). A minimum of 50 metaphases were analyzed per genotype. Arrows indicate fused chromosomes; *indicates signal free chromosome ends. Only a few SFEs are shown. (C) Quantification of percentage of telomere SFEs (left) and chromosome fusions (right) in tumor metaphases shown in (B). Metaphases from lymphomas isolated from at least three different mice were examined. A two‐tailed Student's *t* test was used to calculate statistical significance. (D) Western blot for p53 expression in tumors isolated from mice of the indicated genotype. +: aphidocolin treated WT MEF cells, loaded at 20% the level of other samples. WT: normal splenocytes. γ‐tubulin was used as loading control. (E) Kaplan–Meier survival analysis of mice of the indicated genotypes, monitored over 50 weeks and sacrificed when moribund. The difference in survival between the *Eμ‐myc; Pot1b* ^*Δ/Δ*^ *; Trp53* ^*P/+*^ mice and *Eμ‐myc; Pot1b* ^*Δ/Δ*^ mice was highly significant (*P* < 0.001). (F) Telomere FISH analysis of metaphase chromosome spreads of *Eμ‐myc* and *Eμ‐myc; Pot1b* ^*Δ/Δ*^ *; Trp53* ^*P/+*^ lymphomas using Tam‐OO‐(CCCTAA)_4_ PNA (red) and DAPI (blue). A minimum of 50 metaphases were analyzed per genotype. Arrowheads indicate fused chromosomes; *indicate telomere SFEs. (G) (left) Quantification of percentage of telomere SFEs (left) and chromosome fusions (right) in tumor metaphases shown in (F). Metaphases from lymphomas isolated from at least three different mice were examined. A two‐tailed Student's *t* test was used to calculate statistical significance. (H) Western blot for p53 and p21 expression in tumors isolated from mice of the indicated genotypes. γ‐tubulin was used as loading control.

To ascertain the importance of the p53‐dependent cell cycle arrest/cellular senescence pathway in the suppression of *E*μ*‐myc* lymphomas, we crossed *E*μ*‐myc; Pot1b* ^*∆/∆*^ mice with *p21* ^*−/−*^ mice to generate *E*μ*‐myc; Pot1b* ^*∆/∆*^ *; p21* ^*+/−*^ and *E*μ*‐myc; Pot1b* ^*∆/∆*^ *; p21* ^*−/−*^ mice and monitored the formation of B‐cell lymphomas. Compared with Eμ‐myc; Pot1b^∆/∆^ mice, both *E*μ*‐myc; Pot1b* ^*∆/∆*^ *; p21* ^*+/−*^ and *E*μ*‐myc; Pot1b* ^*∆/∆*^ *; p21* ^*−/−*^ mice displayed significantly reduced tumor‐free survival, although the tumor‐free survival between the two p21 cohorts was not significant (Fig. 4A). This result shows that both alleles of p21 are required to completely repress lymphomagenesis observed in *E*μ*‐myc; Pot1b* ^*∆/∆*^ mice. Telomere PNA‐FISH revealed that compared to *E*μ*‐myc* lymphomas, ~35% of *E*μ*‐myc; Pot1b* ^*∆/∆*^ *; p21* ^*+/−*^ chromosomes displayed telomere‐free ends and ~1.5 end‐to‐end chromosome fusions per metaphase, both indicative of increased telomere dysfunction (Fig. 4B,C). p53 expression was undetectable or aberrant in all *E*μ*‐myc; Pot1b* ^*∆/∆*^ *; p21* ^*+/−*^ lymphomas examined, suggesting that p53 is mutated in these tumors (Fig. 4D). These results reinforce the notion that activation of cell cycle arrest/cellular senescence in *E*μ*‐myc; Pot1b* ^*∆/∆*^ mice by uncapped telomeres is important to suppress B‐cell lymphomagenesis induced by *E*μ*‐myc* oncogene expression.

We next examined how activation of p53‐dependent apoptosis in *E*μ*‐myc; Pot1b* ^*∆/∆*^ mice contributes to the suppression of lymphomagenesis. As indicated previously*, E*μ*‐myc; Pot1b* ^*∆/∆*^ *; p53* ^*+/−*^ mice died by 8 weeks of age from fulminant, infiltrating B‐cell lymphoma, indicating that one allele of WT p53 was insufficient for complete tumor suppression in this genetic setting (Figs 4E and S1A). We next generated *E*μ*‐myc; Pot1b* ^*∆/∆*^ *; p53* ^*R172P/+*^ cohorts (we were unable to generate *E*μ*‐myc; Pot1b* ^*∆/∆*^ *; p53* ^*P/P*^ cohorts despite repeated attempts). Given the tetrameric nature of the p53 complex, a combination of p53^P^ and p53^WT^ proteins will result in only 1 of 16 of the tetramers with fully WT p53 function, and 15 of 16 of the tetramers compromised in p53‐mediated apoptosis (but still competent to mediate cell cycle arrest/cellular senescence). Compared to *E*μ*‐myc; Pot1b* ^*∆/∆*^ mice, *E*μ*‐myc; Pot1b* ^*∆/∆*^ *; p53* ^*R172P/+*^ mice exhibited dramatically reduced tumor‐free survival, with 12 of 12 mice succumbing to B‐cell lymphomas by 11 weeks of age (*P* < 0.001) (Figs 4E and S1B). All *E*μ*‐myc; Pot1b* ^*∆/∆*^ *; p53* ^*R172P/+*^ lymphomas examined displayed dysfunctional telomeres, manifested as telomere‐free chromosome ends involving ~70% of all chromosome ends and ~3 end‐to‐end chromosome fusions (Fig. 4F,G). Western analyses of 9 *E*μ*‐myc; Pot1b* ^*∆/∆*^ *; p53* ^*R172P/+*^ lymphomas revealed that the p53 is aberrantly expressed in 4 of 9 tumors and minimally expressed in the remaining 5. Sequence analysis revealed the presence of mutant p53 allele in 9 of 9 tumors, explaining the loss of the ability to regulate *p21* expression (Fig. 4H and Table 2). Taken together, these results suggest that while complete suppression of *E*μ*‐myc‐*induced lymphomagenesis in the setting of uncapped telomeres requires the activation of both p53‐dependent apoptosis and cellular senescence programs, p53‐dependent apoptosis plays a more prominent role for suppression of B‐cell lymphoma development in *E*μ*‐myc; Pot1b* ^*∆/∆*^ mice.

**Table 2:** DNA sequencing and protein expression of p53 in lymphomas

Mouse number	Mouse genotype	p53 status in lymphoma	p53 protein expression
169	Eμ‐myc	p53^+/+^	Low
594	Eμ‐myc	p53^+/+^	Low
1027	Eμ‐myc	p53^+/+^	Low
1028	Eμ‐myc	p53^+/+^	Low
6033	Eμ‐myc; Pot1b^∆/∆^; p53^p/+^	p53^p/−^	Very high
6007	Eμ‐myc; Pot1b^*∆/∆*^; p53^p/+^	p53^+/−^	Very low
5000	Eμ‐myc; Pot1b^*∆/∆*^; p53^p/+^	p53^p/+^	Very high
6011	Eμ‐myc; Pot1b^*∆/∆*^; p53^p/+^	p53^+/−^	No expression
5320	Eμ‐myc; Pot1b^*∆/∆*^; p53^p/+^	p53^+/−^	No expression
3171	Eμ‐myc; Pot1b^*∆/∆*^; p53^p/+^	p53^p/+^	Very high
2719	Eμ‐myc; Pot1b^*∆/∆*^; p53^p/+^	p53^+/−^	Low
4036	Eμ‐myc; Pot1b^*∆/∆*^; p53^p/+^	p53^+/−^	Low
2098	Eμ‐myc; Pot1b^*∆/∆*^; p53^p/+^	p53^p/+^	Very high

### Telomeres without Pot1b activate p53‐independent apoptosis in highly proliferative organs

**Figure 5:**
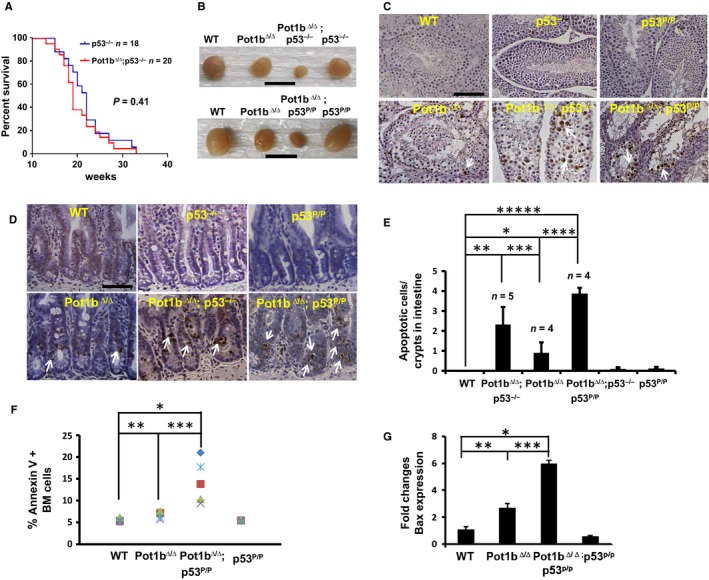
Loss of functional p53 accelerates proliferative defects in *Pot1b* ^*Δ/Δ*^ *; p53* ^*−/−*^ mice. (A) Kaplan–Meier survival analysis showing the percent survival of *p53* ^*−/−*^ and *Pot1b* ^*Δ/Δ*^ *; p53* ^*−/−*^ mice. All mice were monitored for 40 weeks and sacrificed when moribund. The log‐rank test was used to calculate statistical significance. No significant survival difference was observed between the two cohorts (*P* = 0.41). (B) Testes from mice of the indicated genotypes. Scale bar: 10 mm. (C) TUNEL staining of testicular sections from mice of indicated genotypes. Magnification: 20×, scale bar: 50 μm. Representative TUNEL positive cells are indicated by arrowheads. (D) Representative TUNEL stained intestinal sections from mice of the indicated genotypes. Arrowheads indicate apoptotic cells. Scale bar: 25 μm. (E) Quantification of apoptotic cells in basal crypts of intestines from (D). A two‐tailed student's *t* test was used to calculate statistical significance. *P = 4.2 × 10^***−***2^; **P = 4.4 × 10^***−***3^; ***P = 4.6 × 10^***−***2^; ****P = 4.9 × 10^***−***4^; *****P = 4.6 × 10^***−***6^. (F) Annexin V staining of bone marrow cells isolated from indicated mouse cohorts at 30–35 weeks of age. BMs from a minimum of five mice per genotype were analyzed. A two‐tailed student's *t* test was used to calculate statistical significance. *P = 2.1 × 10^−4^; **P = 4.0 × 10^−3^; ***P = 9.0 × 10^−3^. (G) Real‐time RT‐PCR quantification of mRNA expression levels of Bax in sorted LSK cells from 30 to 35 weeks old mice of indicated genotypes. Each experiment was repeated in triplicate. Error bars represent SEM. A two‐tailed student's *t* test was used to calculate statistical significance. *P = 1.21 × 10^−5^; **P = 1.8 × 10^−3^; ***P = 1.0 × 10^−4^.

While chronic activation of p53‐dependent apoptosis and cell cycle arrest/cellular senescence programs by uncapped telomeres is potently tumor suppressive, we have previously shown that an undesirable consequence of *Pot1b* deletion is compromised stem cell proliferation, resulting in systemic multi‐organ failure and the onset of premature aging phenotypes, presumably due to activation of p53‐dependent proliferative checkpoints (Wang *et al*., 2011, 2013). We therefore postulated that deleting p53 in the setting of *Pot1b* deficiency should rescue these adverse phenotypes. Deletion of *Pot1b* did not impact upon the incidence of tumor formation in *p53* ^*−/−*^ mice (Fig. 5A). However, we were surprised to find that compared to age‐matched *Pot1b* ^***∆/∆***^ mice, *Pot1b* ^***∆/∆***^ *; p53* ^*−/−*^ mice exhibited profound cellular proliferative defects in highly proliferative organs, including the testes and intestines. We found that compared to age‐matched *Pot1b* ^***∆/∆***^ mice, both the size and weight of testes from *Pot1b* ^***∆/∆***^ *; p53* ^*−/−*^ mice were significantly reduced (Figs 5B and S4A,B, Supporting information). Testicular weight reduction was even greater when the weights of age‐matched WT and *Pot1b* ^***∆/∆***^ *; p53* ^*−/−*^ testes were compared. Histological analyses revealed that *Pot1b* ^***∆/∆***^ *; p53* ^*−/−*^ testes displayed a complete absence of spermatogenesis in all seminiferous tubules examined. In contrast, most tubules in *Pot1b* ^***∆/∆***^ testes contained germ cells at various developmental stages (Figs 5C, S4C). Analysis of *Pot1b* ^***∆/∆***^ *; p53* ^*P/P*^ testes revealed a nearly identical phenotype of testicular dysfunction (Figs 5C and S4B,C). TUNEL staining revealed significantly increased number of apoptotic cells in both *Pot1b* ^***∆/∆***^ *; p53* ^*−/−*^ and *Pot1b* ^***∆/∆***^ *; p53* ^*P/P*^ testes compared with testes from both WT and *Pot1b* ^***∆/∆***^ mice (Fig. 5C). Examination of the highly proliferative intestinal epithelia by TUNEL and Caspase 3 staining revealed that compared to *Pot1b* ^*∆/∆*^ intestines, *Pot1b* ^***∆/∆***^ *; p53* ^*−/−*^ and *Pot1b* ^***∆/∆***^ *; p53* ^*P/P*^ intestinal crypt epithelia possess significantly increased number of apoptotic cells (Figs 5D,E and S4D). Finally, examination of TBM cells revealed increased apoptosis in cells isolated from *Pot1b* ^*∆/∆*^ *; p53* ^*P/P*^ mice (Figs 5F and S5A, Supporting information). In addition, RT–PCR analysis revealed that BAX expression was increased sixfold in *Pot1b* ^*∆/∆*^ *; p53* ^*P/P*^ cells (Fig. 5G). Together, these results reveal that in addition to the activation of p53‐dependent cell cycle checkpoints, telomeres devoid of Pot1b also activate p53‐independent apoptosis to limit the proliferative capacity of rapidly dividing tissues.

### DNA damage and apoptosis in *Pot1b* ^*∆/∆*^ *; p53* mutant cells requires p73

**Figure 6:**
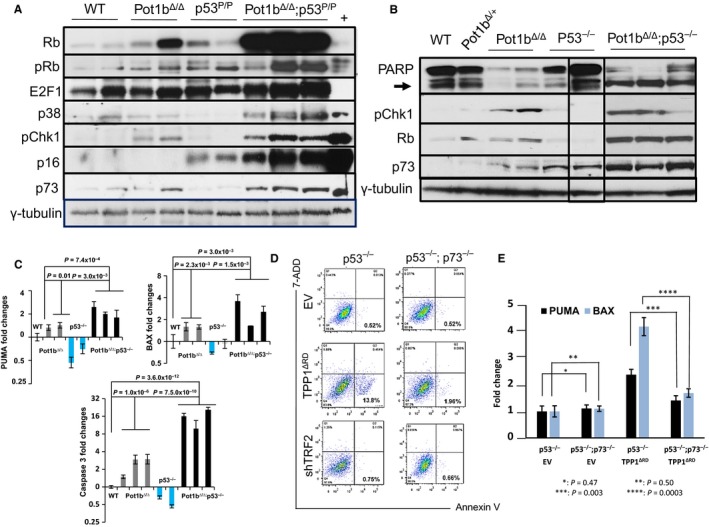
Activation of p53 independent apoptosis in *Pot1b* ^*Δ/*^ *; p53* ^*−/−*^ mice. (A) Immunoblotting for total Rb, phosphorylated‐Rb, E2F1, p38 MAP kinase, phosphorylated‐Chk1, p16 and p73 expression in mouse spleen cells of the indicated genotypes. γ‐tubulin was used as the loading control. (B) Western analysis for PARP, cleaved PARP (arrow), phospho‐Chk1, total Rb and p73 expression in mouse spleen cells of the indicated genotypes. γ‐tubulin was used as the loading control. Samples were run on the same gel but the lanes were not all contiguous. (C) Real‐time RT‐PCR results showing the expression of PUMA (upper left), BAX (upper right) and Caspase 3 (lower) in spleen cells from WT,* Pot1b* ^*Δ/Δ*^ *, p53* ^***−**/**−***^ and Pot1b^*Δ/Δ*^ *; p53* ^***−**/**−***^ mice. Each experiment was repeated in triplicate. Error bars represent SEM. (D) Representative FACS analysis of Annexin V and 7‐AAD expression in *p53* ^***−**/**−***^ and *p53* ^***−**/**−***^ *; p73* ^***−**/**−***^ MEFs 72 h after treatment with empty vector (EV), TPP1^ΔRD^ or shTRF2. *p53* ^*−/−*^ MEFs treated with TPP1^ΔRD^ displayed increased number of Annexin‐V+, 7‐AAD‐ cells, which was significantly reduced in *p53* ^***−**/**−***^ *;p73* ^***−**/**−***^ MEFs. (E) Real‐time (RT)‐PCR analysis of PUMA and BAX expression profiles in *p53* ^***−**/**−***^ and *p53* ^***−**/**−***^ *; p73* ^***−**/**−***^ MEFs after 72 h of treatment with EV or TPP1^ΔRD^. Compared with p53^***−***/***−***^ MEFs expressing ^TPP1ΔRD^, PUMA and BAX expression was largely abrogated in *p53* ^***−**/**−***^ *; p73* ^***−**/**−***^ MEFs expressing TPP1^ΔRD^. Each experiment was repeated in triplicate. Error bars represent SEM. A 2‐way ANOVA analysis was performed to calculate statistical significance.

Rb functions as a tumor suppressor and prevents cell cycle progression by binding to the E2F family of transcription factors. Inactivation of Rb can initiate E2F1‐mediated transactivation of pro‐apoptotic genes, resulting in p53‐independent apoptosis (Nahle *et al*., 2002; Polager & Ginsberg, 2008). Western analysis revealed that both E2F1 and the hypophosphorylated, active form of Rb are highly expressed in *Pot1b* ^***∆/∆***^ *; p53* ^*−/−*^ and *Pot1b* ^***∆/∆***^ *; p53* ^*P/P*^ splenocytes, suggesting that the Rb‐E2F1 pathway is functional in these cells (Fig. 6A,B). This notion is supported by increased p16 expression, which could promote the formation of active Rb‐E2F1 complexes to repress pro‐apoptotic genes. We therefore turned our attention to other pathways that activate p53‐independent apoptosis. In response to DNA damage, the checkpoint kinases Chk1 and Chk2, together with E2F1 and the p38 MAP kinase, all promote the activation and stabilization of the p53‐related tumor suppressor p73 (Sanchez‐Prieto *et al*., 2002; Urist *et al*., 2004). Like p53, p73 plays important roles in DNA damage‐initiated apoptosis (Flores *et al*., 2002, 2005). Western analysis of *Pot1b* ^***∆/∆***^ *; p53* ^*−/−*^ and *Pot1b* ^***∆/∆***^ *; p53* ^*P/P*^ splenocytes revealed robust up‐regulation of Chk1, E2F1, p73, p38 MAP kinase, suggesting that p73 is active in these cells (Fig. 6A,B). The level of transcriptional targets of p73, including BAX, PUMA, and Caspase 3, is also significantly increased at the RNA level in the absence of functional p53 (Fig. 6C). In contrast, anti‐apoptotic genes, including Bcl‐2 and Bcl‐XL, are not induced in Pot1b^***∆/∆***^; p53^*−*/*−*^ splenocytes of following Pot1b deletion (Fig. S5B Supporting information). Importantly, control experiments reveal that expression of p21 was undetectable in *Pot1b* ^*∆/∆*^ *; p53* ^*−/−*^ splenocytes (Fig. S5C Supporting information), suggesting that cellular senescence is not prominently activated in this setting.

Our results suggest that p53‐independent apoptosis is induced in diverse murine cell types by the removal of Pot1b from telomeres. In addition, a recent report revealed that deletion of p53 potently activates p73‐ via E2F‐1‐mediated transcription, suggesting that up‐regulation of p73 in p53^*−*/*−*^ cells could play an important role in mediating p53‐independent apoptosis (Tophkhane *et al*., 2012). To further test this hypothesis, we utilized *p53* ^*−/−*^ and *p53* ^*−/−*^ *; p73* ^*−/−*^ MEFs (Flores *et al*., 2002) to ascertain the role of p73 in p53‐independent apoptosis. We rendered telomeres dysfunctional either by removing Pot1a/b from telomeres using the dominant negative molecule TPP1^ΔRD^ (Deng *et al*., 2009) or by removing the Shelterin component TRF2. In contrast to removal of Pot1a/b, which generates single‐stranded telomeric G‐overhangs and preferentially activates an ATR‐Chk1‐dependent DDR, removal of TRF2 generates chromosome ends resembling double‐stranded DNA breaks and only activates an ATM‐Chk2‐dependent DDR (Denchi & de Lange, 2007; Guo *et al*., 2007; Rai *et al*., 2011). Cellular proliferation, as measured by both low density seeding and NIH 3T3 assays, was compromised in *p53* ^*−/−*^ MEFs expressing TPP1^ΔRD^ (Fig. S6A,B, Supporting information). In contrast, *p53* ^*−/−*^ *; p73* ^*−/−*^ MEFs expressing TPP1^ΔRD^ grew robustly. Deletion of p73 did not compromise TIF formation or number of fused chromosomes generated, indicating that p73 status does not impact upon DNA damage signaling or repair of uncapped telomeres (Fig. S6B,C Supporting information). FACS analysis for 7‐ADD and Annexin V staining revealed that *p53* ^*−/−*^ MEFs expressing TPP1^ΔRD^ exhibited increased apoptosis (13.8%). In contrast, apoptotic cells were observed in only 1.96% of *p53* ^*−/−*^ *; p73* ^*−/−*^ MEFs expressing TPP1^ΔRD^ (Fig. 6D). In support of this result, both Bax and PUMA expression levels, while elevated in *p53* ^*−/−*^ MEFs expressing TPP1^ΔRD^, were reduced to background levels in *p53* ^*−/−*^ *; p73* ^*−/−*^ MEFs (Fig. 6E).

To further ascertain the mechanisms underlying this activation of p73, we examined cellular growth in *p53* ^*−/−*^ and *p53* ^*−/−*^ *; p73* ^*−/−*^ MEFs depleted of TRF2. As shown in Figs 6D,E and S6A,B, depletion of TRF2 did not result in compromised cellular proliferation nor increased apoptosis in either *p53* ^*−/−*^ nor *p53* ^*−/−*^ *; p73* ^*−/−*^ MEFs. Taken together, these results suggest that removal of Pot1b from telomeres preferentially activates a p53‐independent, p73‐dependent apoptotic program to suppress growth.

### p53‐independent apoptosis represses B‐cell, but not T‐cell lymphomagenesis

**Figure 7:**
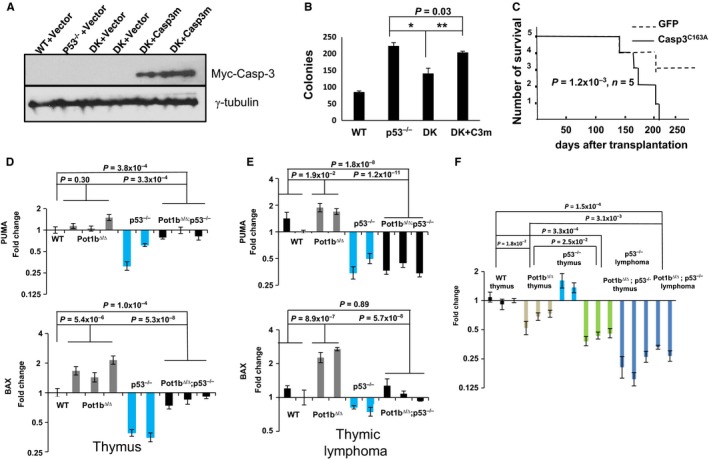
Activation of p53‐independent apoptosis represses B‐cell, but not T‐cell lymphomagenesis. (A) Immunoblotting for Myc‐Caspase‐3^C163A^ expression in FACS sorted GFP+ fetal liver cells of the indicated genotypes. Vector: MSCV‐IRES‐GFP empty vector. Casp3m: MSCV‐Myc‐Caspase‐3^C163A^‐IRES‐GFP. DK:* Pot1b* ^*Δ/Δ*^ *; p53* ^***−**/**−***^ double knockout cells. γ‐tubulin was used as the loading control. (B) Quantification of *in vitro* colony forming assays for sorted GFP+ fetal liver cells in B‐cell specific M3630 medium. Results are mean from three independent experiments. Error bars represent SEM. A two‐tailed student's *t* test was used to calculate statistical significance*P = 2.1 × 10^−3^; **P = 3.1 × 10^−3^. (C) Kaplan–Meier survival analysis showing the percent survival of SCID mice transplanted with either *Pot1b* ^*Δ/Δ*^ *; p53* ^***−**/**−***^ fetal liver cells expressing empty vector or MSCV‐Myc‐Caspase‐3^C163A^‐IRES‐GFP. (D–G) Real‐time RTPCR analysis of PUMA and BAX expression profiles in thymus (D) and thymic lymphomas (E) from 6 weeks old mice of the indicated genotypes. Each experiment was repeated in triplicate. Error bars represent SEM. A 2‐way ANOVA analysis was performed to calculate statistical significance. (F) Real‐time RT‐PCR analysis of p73 expression profiles in 6 weeks thymus and thymic lymphomas of the indicated genotypes. Each experiment was repeated in triplicate. Error bars represent SEM. A 2‐way ANOVA analysis was performed to calculate statistical significance.

Previously reports reveal that p73 is able to activate Caspase 3 to induce apoptosis (Cottini *et al*., 2014). We found that Caspase 3‐mediated cleavage of PARP‐1 was readily detected in *Pot1b* ^*∆/∆*^ *; p53* ^*−/−*^ splenocytes, indicative of robust p53‐independent apoptosis (Fig. 6B). The observation of increased p‐Chk1 levels in *Pot1b* ^***∆/∆***^ *; p53* ^*P/P*^ and *Pot1b* ^*∆/∆*^ *; p53* ^*−/−*^ splenocytes, but not in WT or *p53* ^*−/−*^ splenocytes, suggests that apoptosis was induced by dysfunctional telomeres eliciting an ATR‐Chk1‐dependent DDR. To confirm functionally that activation of Caspase 3 is important to limit proliferation of *Pot1b* ^*∆/∆*^ *; p53* ^*−/−*^ splenocytes, we expressed a dominant negative Caspase 3 mutant (MSCV‐Caspase 3^C163A^‐Myc–GFP) (Huang *et al*., 2011) in WT, *p53* ^*−/−*^ and *Pot1b* ^*∆/∆*^ *; p53* ^*−/−*^ fetal liver stem cells and FACS sorted for cells robustly expressing mutant Caspase 3. The sorted cells were then cultured in media to specifically promote pre‐B‐cell differentiation and proliferation. Western analysis confirmed Caspase 3^C163A^‐Myc expression after 12 days of culture (Fig. 7A). Expression of Caspase 3^C163A^ significantly improved the ability of *Pot1b* ^*∆/∆*^ *; p53* ^*−/−*^ pre‐B cells to form colonies *in vitro*, suggesting that p53‐independent activation of Caspase 3 limited B‐cell proliferation (Fig. 7B). In support of this notion, transplantation of *Pot1b* ^*∆/∆*^ *; p53* ^*−/−*^ hematopoietic cells expressing Caspase 3^C163A^‐Myc into SCID mice resulted in significantly increased induction of B‐cell lymphomas, suggesting that p53‐independent apoptosis normally represses lymphomagenesis through the up‐regulation of Caspase 3 (Fig. 7C). To strengthen this observation, we examined the impact that activation of p53‐independent apoptosis has on spontaneous tumorigenesis in our mouse cohorts. We found that while our cohort of p53^*−*/*−*^ mice normally succumbed to B‐cell lymphomas derived from lymph nodes and spleens, a dramatic shift in tumor type to thymic lymphomas was observed in *Pot1b* ^*∆/∆*^ *; p53* ^*−/−*^ mice (Table 3). RT–PCR analysis revealed that in contrast to the high levels of PUMA and Bax transcripts observed in *Pot1b* ^*∆/∆*^ *; p53* ^*−/−*^ splenocytes, both transcripts were significantly reduced in thymocytes and in thymic lymphomas (Figs 6C–E and 7D,E). We also found that p73 expression was markedly reduced in thymic lymphomas (Fig. 7F). Taken together, these results suggest that activation of p53‐independent, p73‐dependent apoptosis in *Pot1b* ^*∆/∆*^ *; p53* ^*−/−*^ mice limited B‐cell proliferation to suppress B‐cell lymphomas but did not suppress the formation of thymic lymphomas.

**Table 3:** Tumor profile of p53^−/^^−^ and Pot1b^Δ/^ ^Δ^; p53^−/^^−^ mice

	p53^−/−^	Pot1b^Δ/Δ^; p53^−/−^	*P* value
Lymphoma total	11	13	0.80
Thymic lymphomas	3	10	0.01
Other tumors	6	4	0.35
No gross tumors	1	4	0.19 (Chi‐square test)
Total	18	20	

## Discussion

The incidence of human cancers is inextricably linked with advancing age. Increased DNA damage and accumulation of somatic mutations is the major driving force behind cancer development in the elderly. Mutations in p53 involve more than half of all human cancers, highlighting p53′s importance in tumor suppression. Understanding mechanistically how induction of p53 suppresses tumor formation is therefore of significant interest in an increasingly aged population. Here, we use an established mouse model of Burkett's B‐cell lymphoma to show that dysfunctional telomeres devoid of the shelterin component Pot1b potently activate a p53‐dependent DDR to almost completely suppress Myc oncogene‐driven B‐cell lymphomagenesis. By generating *E*μ*‐myc; Pot1b* ^*∆/∆*^ *; p53* ^*P/+*^ mice compromised in the induction of p53‐dependent apoptosis and *E*μ*‐myc; Pot1b* ^*∆/∆*^ *; p21* ^*−/−*^ mice unable to initiate p53‐dependent cellular senescence, we are able to directly compare the tumor suppressive contributions of p53‐mediated apoptosis vs. cellular senescence. While we and others have previously shown that activation of the p53‐dependent senescence program by dysfunctional telomeres is critical to suppress tumorigenesis (Cosme‐Blanco *et al*., 2007; Feldser & Greider, 2007), this is the first time that the effects of telomere‐induced senescence and apoptosis are directly compared side by side in mouse littermates. Our results indicate that while cooperation of both programs is required to fully suppress oncogene‐induced lymphomagenesis, dysfunctional telomere‐induced apoptosis plays a more important role than cellular senescence in suppressing tumorigenesis. In addition, our studies uncover a role for the p53‐ortholog p73 in mediating tumor suppression in the absence of p53. Telomeres devoid of Pot1b are able to activate a p53‐independent, p73‐dependent apoptotic program to prevent B‐cell lymphoma development. The data presented here highlight the various p53‐dependent and independent antiproliferative mechanisms employed by mammalian cells to combat dysfunctional telomere‐induced genomic instability and the generation of lymphomas.

The p53 family members p63 and p73 share many features with p53, including transcriptionally regulating a number of genes in response to DNA damage (Yang & McKeon, 2000; Irwin & Kaelin, 2001; Lin *et al*., 2009). Unlike p53, both p63 and p73 are structurally complex, with multiple isoforms functioning both as oncogenes and tumor suppressors. For example, the p73 gene has two promoters that results in the generation of transactivating (TA) and the ΔN form of p73 (Yang *et al*., 1998; Grob *et al*., 2001). Both the TA and ΔN isoforms of p73 can transactivate target genes to induce apoptosis and growth arrest following DNA damage (Irwin *et al*., 2003; Liu *et al*., 2004a). Importantly, in the absence of p53, p73 isoforms are able to induce apoptosis and growth arrest (Willis *et al*., 2003; Lin *et al*., 2009; Tophkhane *et al*., 2012). While p53‐dependent apoptosis is highly tumor suppressive in our mouse lymphoma models, we found that highly proliferative tissues from *Pot1b* ^*∆/∆*^ *; p53* ^*−/−*^ mice also displayed robust TUNEL staining and activation of pro‐apoptotic factors, including BAX, PUMA, and Caspase 3. This p53‐independent, p73‐dependent apoptosis was able to inhibit spontaneous B‐cell lymphomagenesis normally observed in *p53* ^*−/−*^ mice. Transplantation of *Pot1b* ^*∆/∆*^ *; p53* ^*−/−*^ B cells expressing a dominant negative form of Caspase 3 promotes lymphomagenesis in SCID mice, further supporting the hypothesis that dysfunctional telomeres induced by Pot1b loss activates p53‐independent apoptosis to suppress the formation of B‐cell lymphomas. However, p53‐independent apoptosis induced by *Pot1b* deletion also compromises organ homeostasis, promoting the onset of premature age‐related phenotypes.

We have previously shown that in the setting of *Pot1b* deficiency, increased generation of single‐stranded telomeric DNA promotes the activation of the ATR‐Chk1 checkpoint kinases (Guo *et al*., 2007; He *et al*., 2009; Flynn *et al*., 2011; Thanasoula *et al*., 2012). We now propose a model in which telomeres devoid of Pot1b activates an ATR‐dependent DNA damage response (DDR), activation of Chk1 stabilizes E2F1, and both proteins transcriptionally activate p73 (Sanchez‐Prieto *et al*., 2002; Urist *et al*., 2004; Tophkhane *et al*., 2012) (Fig. S7, Supporting information). Consistent with its p53‐independent tumor suppressive function, the levels of p73 targets including BAX, PUMA, and Caspase 3 are significantly increased in *Pot1b* ^*∆/∆*^ *; p53* ^*−/−*^ spleens Pot1b deficient telomeres induce p73 to activate BAX and PUMA, as the expression of both of these pro‐apoptotic genes is abrogated in *p53* ^*−/−*^ *; p73* ^*−/−*^ MEFs. Activation of p73 serves to restrain the progression toward cancer in hematopoietic tissues. However, it also induced undesirable effects, including premature onset of age‐related phenotypes. That an ATR‐Chk1‐dependent DDR may be required to potently activate p53‐independent, p73‐dependent apoptosis is supported by our observation that telomeres devoid of TRF2, which activates an ATM‐Chk2‐dependent DDR (Rai *et al*., 2010), did not induce apoptosis in either *p53* ^*−/−*^ or *p53* ^*−/−*^ *; p73* ^*−/−*^ MEFs. p73 has been shown to upregulate several DNA repair genes, including Rad51, BRCA2, and MRE11, and these are also the same ones needed to repair dysfunctional telomeres lacking functional Pot1a/b proteins (Lin *et al*., 2009; Rai *et al*., 2010). Taken together, our data suggest that initiation of p73‐dependent apoptosis may be uniquely dependent upon the activation of distinct types of DNA damage responses and repair pathways in precursor cancer cells.

Interestingly, p53‐independent apoptosis was observed to preferentially limit the development of B‐cell but not in thymic lymphomas, resulting in decreased formation of B‐cell lymphomas and an increase in the generation of spontaneous thymic lymphomas in *Pot1b* ^*∆/∆*^ *; p53* ^*−/−*^ mice. This tissue‐specific activation of BAX and PUMA by telomeres devoid of Pot1b in B cells but not in T cells is not currently understood. We speculate that the ATR‐Chk1‐dependent DDR pathway is not activated in *Pot1b* ^*∆/∆*^ *; p53* ^*−/−*^ T cells, resulting in lymphomagenesis.

Expression of mutant forms of the telomerase protein or the shelterin component TIN2 is able to promote cell death in a p53‐independent manner (Hahn *et al*., 1999; Guiducci *et al*., 2001; Kim *et al*., 2008). In addition, telomerase null mice experience increased sterility as their telomeres shorten, and this phenotype is not recused by deleting p53 (Chin *et al*., 1999). Because perturbation of telomere homeostasis in *p53* ^*−/−*^ cells and in late generation mTerc^*−*/*−*^; p53^*−*/*−*^ mice results in increased chromosomal fusions, it is generally believed that it is this elevated genomic instability that results in eventual cell death (Begus‐Nahrmann *et al*., 2009; Sperka *et al*., 2012b) (Fig. S7). Our data suggest that in addition to elevated chromosomal instability, dysfunctional telomeres lacking Pot1b also activate an ATR‐Chk1 DDR pathway to induce p53‐independent, p73‐dependent apoptosis. Activation of p73 has been recently shown to restrain tumorigenesis in the absence of functional p53, suggesting that this could represent a novel strategy to specifically target p53‐deficient tumors (Venkatanarayan *et al*., 2015).

## Materials and methods

### Mice

*E*μ*‐myc*,* Pot1b* ^Δ*/*Δ^, *E*μ*‐myc*;* Pot1b* ^Δ*/*Δ^, *Pot1b* ^Δ*/*Δ^; *p21* ^*−/−*^, *Pot1b* ^Δ*/*Δ^; *p53* ^*−/−*^, *Pot1b* ^Δ*/*Δ^; *p53* ^*P/P*^ mice were generated and maintained according to the IACUC‐approved protocols of Yale University (Harris *et al*., 1988; Liu *et al*., 2003; He *et al*., 2009; Wang *et al*., 2010; Sperka *et al*., 2012a). To generate *E*μ*‐myc; Pot1b* ^*Δ/Δ*^ mice, we crossed *E*μ*‐myc* mice with *Pot1b* ^*Δ/+*^ mice to get *E*μ*‐myc; Pot1b* ^*Δ/+*^ mice. We then crossed *E*μ*‐myc; Pot1b* ^*Δ/+*^ mice with *Pot1b* ^*Δ/+*^ mice to get *E*μ*‐myc; Pot1b* ^*Δ/Δ*^ mice. To generate *E*μ*‐myc; Pot1b* ^*Δ/Δ*^ *; p53* ^*−/−*^ mice, we crossed *E*μ*‐myc; Pot1b* ^*Δ/*+^ mice with *Pot1b* ^*Δ/+*^ *; p53* ^*+/−*^ or *Pot1b* ^*Δ/+*^ *; p53* ^*−/−*^ mice to get *E*μ*‐myc; Pot1b* ^*Δ/Δ*^ *; p53* ^*+/−*^ mice. We then crossed *E*μ*‐myc; Pot1b* ^*Δ/Δ*^ *; p53* ^*+/−*^ mice with *Pot1b* ^*Δ/Δ*^ *; p53* ^*+/−*^ mice to get *E*μ*‐myc; Pot1b* ^*Δ/Δ*^ *; p53* ^*−/−*^ mice. To generate *E*μ*‐myc; Pot1b* ^*Δ/Δ*^ *; p53* ^*P/+*^ mice, we crossed *E*μ*‐myc; Pot1b* ^*Δ/+*^ mice with *Pot1b* ^*Δ/+*^ *; p53* ^*P/P*^ mice to get *E*μ*‐myc; Pot1b* ^*Δ/Δ*^ *; p53* ^*P/+*^ mice. We then crossed *E*μ*‐myc; Pot1b* ^*Δ/Δ*^ *; p53* ^*P/+*^ mice with *Pot1b* ^*Δ/Δ*^ *; p53* ^*P/+*^ mice to get *E*μ*‐myc; Pot1b* ^*Δ/Δ*^ *; p53* ^*P/+*^ mice. To generate *E*μ*‐myc; Pot1b* ^*Δ/Δ*^ *; p21* ^*+/−*^ and *E*μ*‐myc; Pot1b* ^*Δ/Δ*^ *; p21* ^*−/−*^ mice, we crossed *E*μ*‐myc; Pot1b* ^*Δ/+*^ mice with *Pot1b* ^*Δ/+*^ *; p21* ^*−/−*^ mice to get *E*μ*‐myc; Pot1b* ^*Δ/Δ*^ *; p21* ^*+/−*^ and *E*μ*‐myc; Pot1b* ^*Δ/+*^ *; p21* ^*+/−*^ mice. We then crossed *E*μ*‐myc; Pot1b* ^*Δ/+*^ *; p21* ^*+/−*^ mice with *Pot1b* ^*Δ/+*^ *; p21* ^*−/−*^ mice to get *E*μ*‐myc; Pot1b* ^*Δ/Δ*^ *; p21* ^*−/−*^ mice.

### Histology and TUNEL assay

Tissues were fixed in 10% formalin, paraffin embedded, sectioned at 5 μm thickness and stained with H&E. The terminal deoxynucleotidyltransferase‐mediated dUTP‐biotin nick end labeling (TUNEL) assay was performed using the ApopTag Plus peroxidase *in situ* apoptosis detection kit (Chemicon, 28820 Single Oak Drive, Temecula, CA 92590, USA) according to the manufacturer's instructions.

### Flow cytometry analysis

To detect lineage markers on the bone marrow (BM) cells, 1 × 10^7^ BM cells were centrifuged and resuspended in 200 μL of HBSS^+^ (Invitrogen, 542 Flynn Road, Camarillo CA 93012, USA), then stained for 15 min with antibodies. After staining, cells were washed, resuspended in HBSS^+^, and analyzed by flow cytometry (LSRII; BD, 2350 Qume Drive San Jose, CA 95131, USA). For B‐cell sorting, cells were stained with B220 (RM‐2601; Caltag, 5791 Van Allen Way Carlsbad, CA 92008 USA) and IgM (Caltag). For LSK cell staining, a cocktail of antibodies including nine lineage markers was used as follows: Ter‐119, CD3, CD4, CD8, B220, CD19, IL‐7Rα, GR‐1, and Mac‐1 (eBioscience, 10255 Science Center Dr, San Diego, CA 92121, USA) conjugated with APC‐Cy‐7 (47‐4317; eBioscience). The cocktail also contained anti‐Sca‐1‐PE (12‐598; eBioscience) and anti‐c‐Kit‐APC (17‐1171; eBioscience). LSK populations were selected based on low or negative expression of the mature lineage markers and dual‐positive expression for Sca‐1 and c‐Kit. To determine the proliferative status of LSK cells, BrdU was injected intraperitoneally into mice at a dose of 150 mg/kg body weight 2 h before euthanization. Analysis of BrdU incorporation was performed using the FITC BrdU Flow Kit (BD). To quantitatively determine the percentage of cells that were actively undergoing apoptosis, the Annexin V‐PE Apoptosis Detection Kit (BD Pharmingen, 2350 Qume Drive San Jose, CA 95131, USA) was used.

### Isolation of total BM cells and colony‐forming assay

Hindlimb bones were dissected, and the marrow was flushed through a 21‐gauge needle into HBSS^+^, 2% FBS (Invitrogen), and 10 mM HEPES. The cells were passed through a 25‐gauge needle twice and filtered (40‐μm filter, BD Falcon^™^, BD Biosciences, 2350 Qume Drive San Jose, CA 95131 ) to ensure a single‐cell suspension. Nucleated cells were counted manually after the lysis of RBCs with 3% acetic acid in methylene blue (STEMCELL Technologies, 570 West Seventh Avenue, Suite 400, Vancouver, BC, V5Z 1B3, Canada). For the colony‐forming assay, 1 × 10^4^ BM mononucleated cells (BM MNCs) or 3 × 10^4^ GFP+ cells were cultured in 35‐mm dishes containing MethylCult 3630 (STEMCELL Technologies) following the manufacturer's protocols. The colonies were counted on day 8.

### PNA‐FISH and TIF and immunofluorescence‐FISH assays

Cells were treated with 0.5 μg/mL of colcemid for 2–4 h before harvest. Metaphase chromosomes from BM were prepared 1–2 h after colcemid treatment and subjected to telomere peptide nucleic acid FISH staining to label telomeres. Depending on the quality of metaphase spreads, 20–50 metaphases from each sample were analyzed in detail. Cells were treated with 0.6M KCl, fixed with methanol: acetic acid (3:1), and spread on glass slides. Metaphase spreads were hybridized with 5′‐Tam‐OO‐(CCCTAA) 4‐3′ probe (Panagene, 54, Techno 10‐ro Yuseong‐gu Daejeon, Korea). For the TIF assay, cells were seeded in the 8‐well chambers and immunostained with primary antibody and FITC‐secondary antibody, then hybridized with 5′‐Tam‐OO‐(CCCTAA) 4‐3′ probe. TIF analysis in LSK cells and B220 + cells was performed as previously described (Wang *et al*., 2011). All microscopy was performed with a Nikon Eclipse 80i and an Andore CCD camera utilizing Metamorph software.

### Reverse transcriptase coupled RT–PCR

Total RNA was prepared using QIAGEN's RNeasy Micro kit (QIAGEN, 27220 Turnberry Lane Suite 200 Valencia, CA 91355) according to the manufacturer's instructions. For first‐strand cDNA synthesis, 1 μg of total RNA, 20 pmol of Oligo (dT)12–18, and 200 units of SuperScript II Reverse Transcriptase (Invitrogen) were mixed in a final volume of 20 μL. Synthesized cDNA (1 μL) was added to a 20‐μL PCR mixture containing TaqMan Gene Expression Assay primers (Applied Biosystems, 850 Lincoln Centre Drive Foster City, CA 94404, USA.) and TaqMan Universal PCR Master Mix. Each sample was amplified in triplicate. PCR consisted of 40 cycles of denaturation at 95 °C for 15 s, annealing, and amplification at 60 °C for 60 s in an ABI7900HT Sequence Detection System machine (Applied Biosystems). The specific primers for the TaqMan Gene Expression Assay primers were as follows: PUMA: Mm00519268_m1; BAX: Mm00432050_m1; p21:Mm00432448_ml; FasL: Mm00438864_ml; Caspase 3: Mm01195085_ml. p16: Mm01257348_m1 p73:Mm00660220_ml. Bcl2:Mm00477631_m1. Bcl‐xL:Mm00437783_m1. 18S rRNA primers (4319413E or Mm00519571_ml) were used as internal controls.

### Western blot analysis

The antibodies used for Western blot analysis were as follows: phospho‐p53 (Ser15) (Cell Signaling Technology, 3 Trask Lane Danvers, MA 01923, USA; 1:500); phospho‐Chk1 (Cell Signaling Technology; 1:500), anti‐mouse p21 (sc‐397; Santa Cruz Biotechnology Inc., 10410 Finnell Street, Dallas, Texas 75220, USA,1:500), anti‐phospho‐Rb (sc‐56174; Santa Cruz Biotechnology Inc., 1:500), antitotal Rb (sc‐74562; Santa Cruz Biotechnology Inc., 1:500), anti‐E2F1 (sc‐251; Santa Cruz Biotechnology Inc., 1:500), anti‐p38 (sc‐535; Santa Cruz Biotechnology Inc., 1:500), anti‐p73 (sc‐7957; Santa Cruz Biotechnology Inc., 1:500), anti‐Myc (Millipore, 290 Concord Road, Billerica, MA 01821, USA; 1:500), anti‐PARP (Cell signaling 1:500), and anti‐p16 (Abcam, 1 Kendall Square, Suite B2304, Cambridge, MA 02139, USA, 1:500). Anti‐mouse γ‐tubulin (Sigma‐Aldrich, PO Box 14508, St. Louis, MO 63178, USA; 1:10 000) was used as a loading control.

### Plasmids

The pcDNA3‐Casp3^C163A^‐myc plasmid 11814 was purchased from Addgene. The Casp3C^163A^‐myc insert was subcloned into the BamHI site of MSCV‐IRES‐EGFP (MIG) vector, designated as MSCV‐Caspase 3^C163A^‐Myc–GFP. Retrovirus production and infection method were performed as described previously (Wang *et al*., 2011).

### MEFs culture and proliferation assays

The 3T3 proliferation assays for MEFs were performed following standard protocols. For colony‐forming assay, 1 × 10^3^ cells were seeded in 6‐well tissue culture plates and cultured for 1 week. Colonies were fixed with ice‐cold methanol and stained with crystal violet (0.5% in 25% methanol).

### Statistics

Statistical analyses were performed using GraphPad Prism, version 6 software (GraphPad Software Inc., 7825 Fay Avenue, Suite 230, La Jolla, CA 92037, USA) or Microsoft Excel. Data sets were compared using ANOVA or the Student's *t‐*test (unpaired, 2‐tailed). Kaplan–Meier survival analyses were performed using the log‐rank test. *P* values of 0.05 or less were considered significant.

### Study approval

The animal care and use program at Yale University maintains full accreditation from the Association for Assessment and Accreditation of Laboratory Animal Care (AAALAC) and complies with U.S. Animal Welfare Regulations, the National Research Council (NRC) Guide for the Care and Use of Laboratory Animals, and Public Health Service Policy on Humane Care and Use of Laboratory Animals. Yale University has an approved Animal Welfare Assurance (#A3230‐01) on file with the NIH Office for Protection from Research Risks. The Assurance was approved May 16, 2014. My mouse personnel have multiple years of experience working with mice. Our veterinarian Dr. Peter Smith is always available should health concerns arise. My animal protocols have been approved by the animal care and use program at Yale University (2010‐11358) for 3 years and renewed annually.

## Author contributions

YW and SC conceived the project. YW and XW conducted the experiments, and ERF supplied essential reagents. JY and SC interpreted the data. YW and SC wrote the paper.

## Funding

This work was supported by U01DK085570 and ACS RGS‐10‐124‐01‐CCE to JY, R01CA134796, R01CA160394, and CPRIT 140271 to E.R. F., and RO1 CA129037, RO1CA202816, R21CA200506, R21CA202816, and CT 2015‐0901 to S.C.

## Conflicts of interest

The authors declare no conflict of interests.

## Supporting information


**Fig. S1.** Representative images of (A) *E*μ*‐myc; Pot1b* ^∆/∆^; *p53* ^+/−^ and (B) *E*μ*‐myc; Pot1b* ^∆/∆^ *; p53* ^P/+^ mice.
**Fig. S2.** (A) Images of WT, Pot1b^∆/∆^, Pot1b^∆/∆^; p53^P/P^ and Pot1b^∆/∆^; p53^−/−^ mice at the indicated ages. (B) SA‐β‐galactosidase staining and (C) quantification of SA‐β‐galactosidase positive cells in the bone marrows of mice of the indicated genotypes. (D) Western analysis for p21expression in mouse kidney cells. γ‐tubulin was used as the loading control.
**Fig. S3.** (A) Quantification of total BM nucleated cell counts in 30–35 weeks old mice of the indicated genotypes. (B) Representative FACS analysis of Sca‐1 and C‐kit positive cell populations in 30–35 weeks old mouse BMs of the indicated genotypes.
**Fig. S4.** (A) Quantification of testicular weights from 20 weeks old mice of the indicated genotypes. (B) Quantification of testicular weights from 30 to 35 weeks old mice of the indicated genotypes. (C) H&E stained testicular sections from mice of the indicated genotypes. (D) Representative photographs of Caspase 3 staining of intestinal sections.
**Fig. S5.** (A) Representative histograms showing Annexin V profiles of mouse bone marrow cells isolated from 30 to 35 week old mice of the indicated genotypes. (B) Real‐time RT‐PCR analysis of Bcl2 and BCL‐xL expression profiles in spleenocytes from mice of the indicated genotypes.
**Fig. S6.** (A) Left panel: Representative image of colony forming assays for p53^−/−^ and p53^−/−^; p73^−/−^ MEFs 72 h after treatment with empty vector (EV), TPP1^ΔRD^ or shTRF2. (B) Left panel: Immunostaining for γ‐H2AX‐positive dysfunctional telomere‐induced DNA damage foci (TIFs) following 72 h expression of TPP1ΔRD in p53^−/−^ or p53^−/−^; p73^−/−^ MEFs. (C) Left panel: Telomere PNA‐FISH of metaphase spreads showing end‐to‐end chromosome fusions (arrows).
**Fig. S7.** Model of genetic interactions discussed in the test.
