# Supplementary material for: Dysfunctional telomeres induce p53‐dependent and independent apoptosis to compromise cellular proliferation and inhibit tumor formation
Source: Aging Cell. 2016 Apr 26;15(4):646–60. doi: 10.1111/acel.12476 (PMC4933665; doi:10.1111/acel.12476)
Supplement: Supplementary file 1 — Fig. S1. Representative images of (A) Eμ‐myc; Pot1b ∆/∆; p53 +/− and (B) Eμ‐myc; Pot1b ∆/∆ ; p53 P/+ mice. Fig. S2. (A) Images of WT, Pot1b∆/∆, Pot1b∆/∆; p53P/P and Pot1b∆/∆; p53−/− mice at the indicated ages. (B) SA‐β‐galactosidase staining and (C) quantification of SA‐β‐galactosidase positive cells in the bone marrows of mice of the indicated genotypes. (D) Western analysis for p21expression in mouse kidney cells. γ‐tubulin was used as the loading control. Fig. S3. (A) Quantification of total BM nucleated cell counts in 30–35 weeks old mice of the indicated genotypes. (B) Representative FACS analysis of Sca‐1 and C‐kit positive cell populations in 30–35 weeks old mouse BMs of the indicated genotypes. Fig. S4. (A) Quantification of testicular weights from 20 weeks old mice of the indicated genotypes. (B) Quantification of testicular weights from 30 to 35 weeks old mice of the indicated genotypes. (C) H&E stained testicular sections from mice of the indicated genotypes. (D) Representative photographs of Caspase 3 staining of intestinal sections. Fig. S5. (A) Representative histograms showing Annexin V profiles of mouse bone marrow cells isolated from 30 to 35 week old mice of the indicated genotypes. (B) Real‐time RT‐PCR analysis of Bcl2 and BCL‐xL expression profiles in spleenocytes from mice of the indicated genotypes. Fig. S6. (A) Left panel: Representative image of colony forming assays for p53−/− and p53−/−; p73−/− MEFs 72 h after treatment with empty vector (EV), TPP1ΔRD or shTRF2. (B) Left panel: Immunostaining for γ‐H2AX‐positive dysfunctional telomere‐induced DNA damage foci (TIFs) following 72 h expression of TPP1ΔRD in p53−/− or p53−/−; p73−/− MEFs. (C) Left panel: Telomere PNA‐FISH of metaphase spreads showing end‐to‐end chromosome fusions (arrows). Fig. S7. Model of genetic interactions discussed in the test. [file ACEL-15-646-s001.pdf]

A

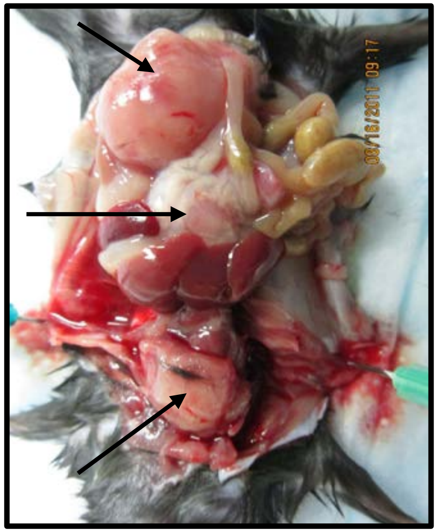

Eμ-myc;Pot1b$^{\Delta/\Delta}$;p53$^{+/-}$

B

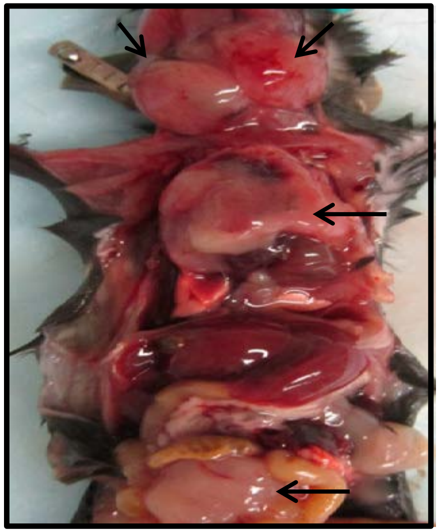

Eμ-myc;Pot1b$^{\Delta/\Delta}$;p53$^{P/+}$

Supplementary Figure 1

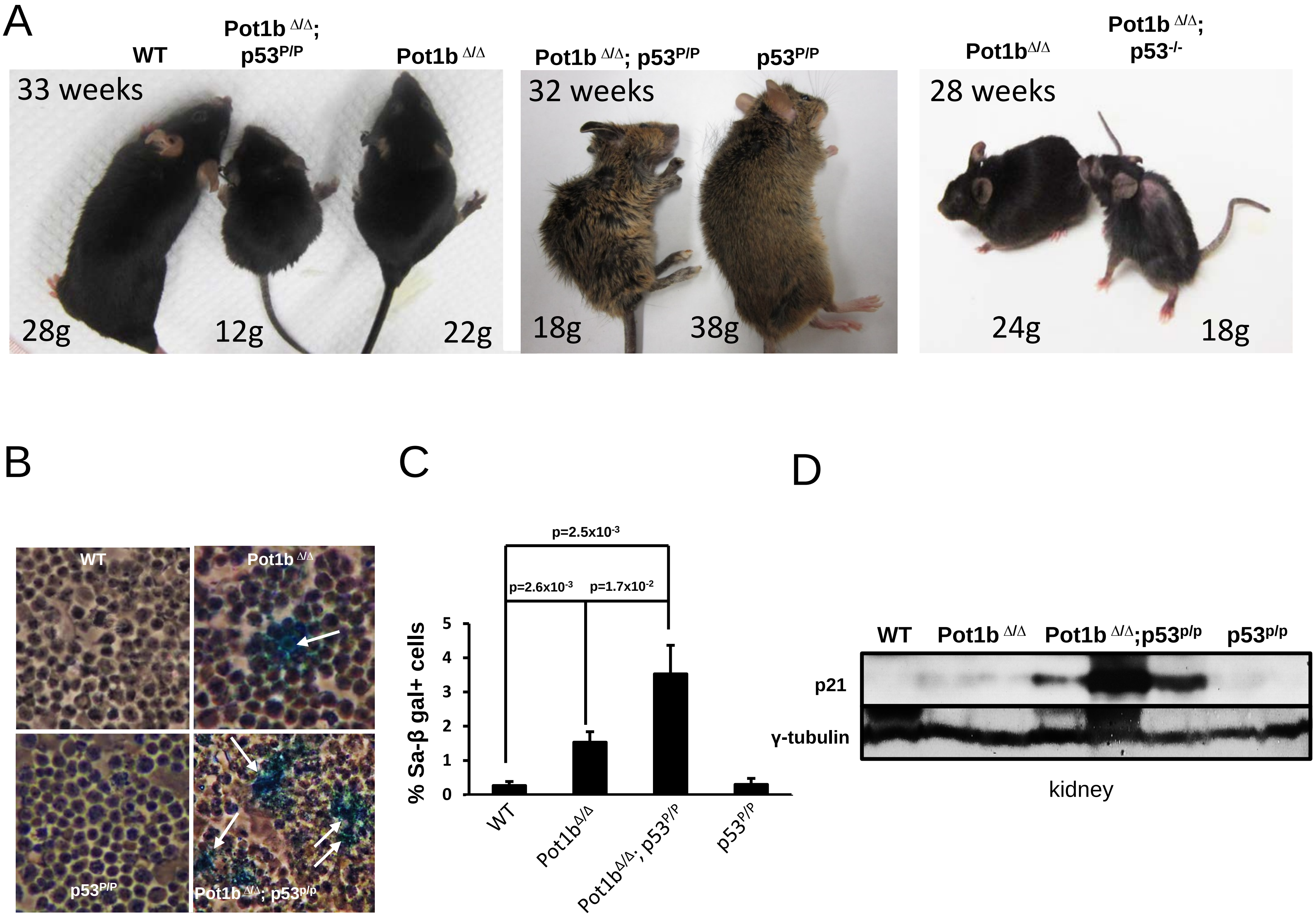

Supplementary Figure 2

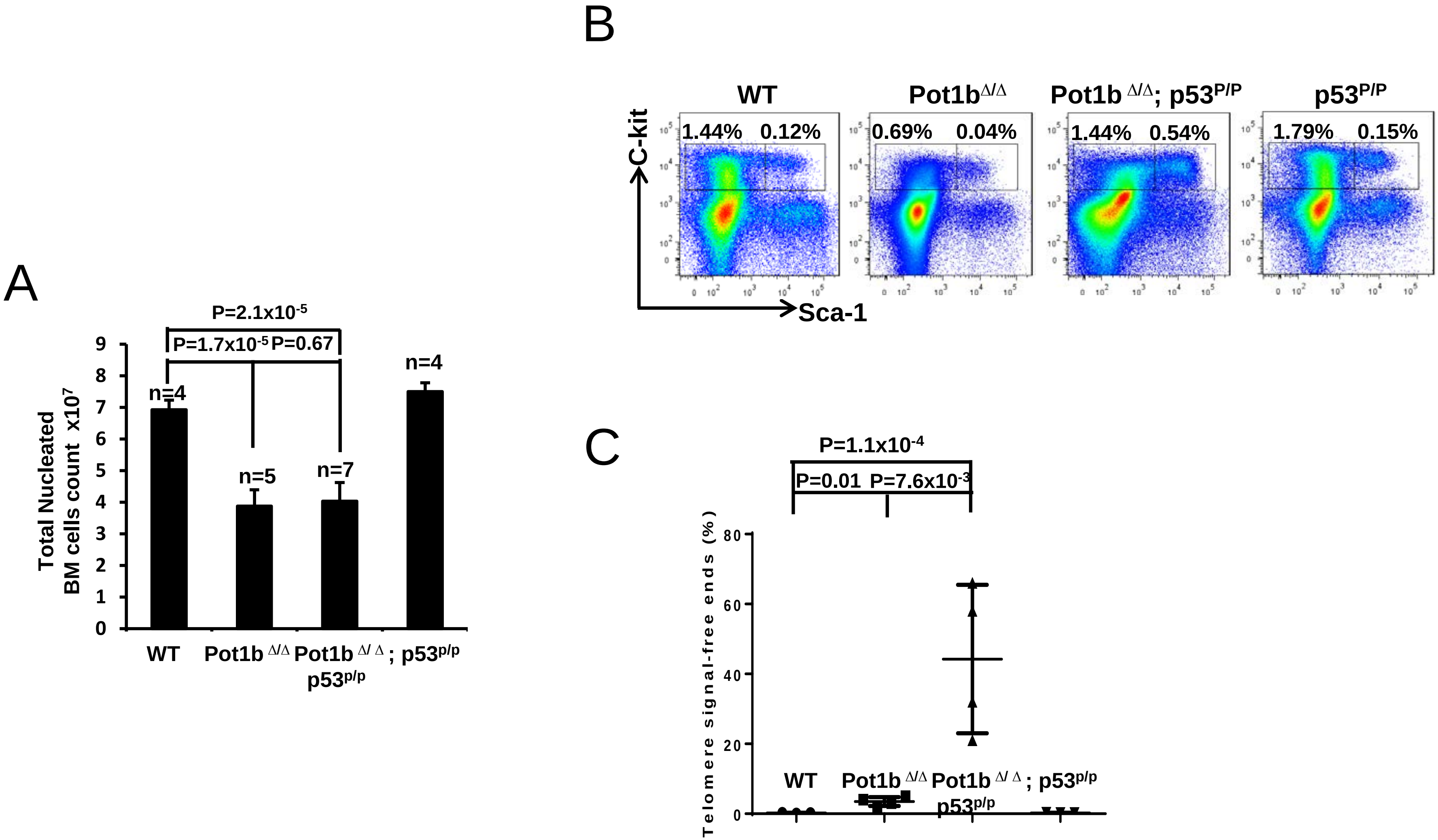

Supplementary Figure 3

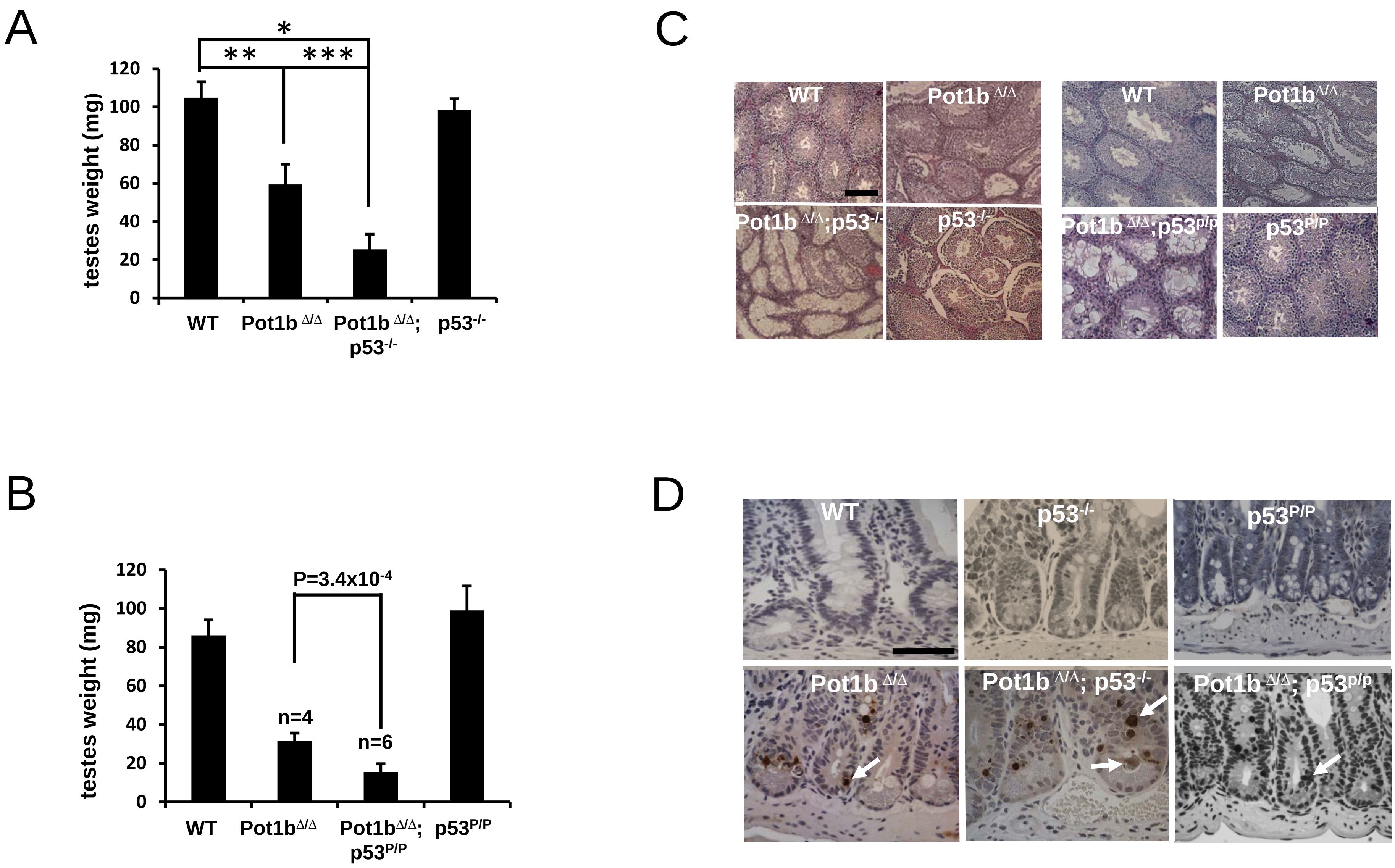

Supplementary Figure 4

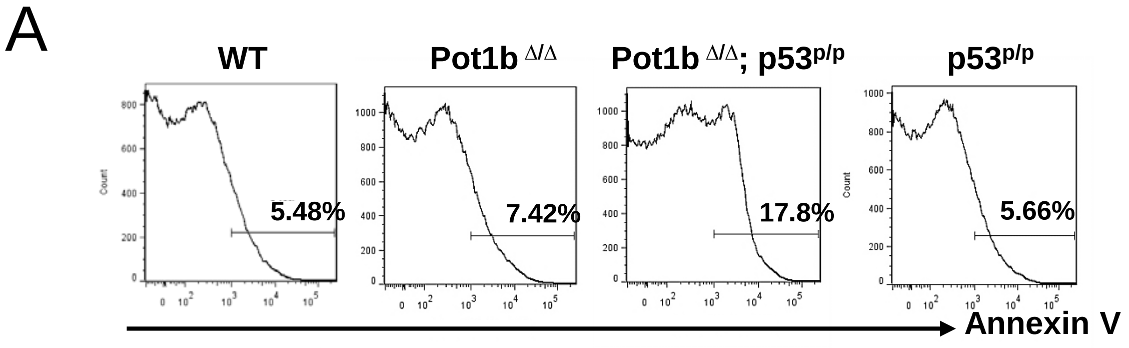

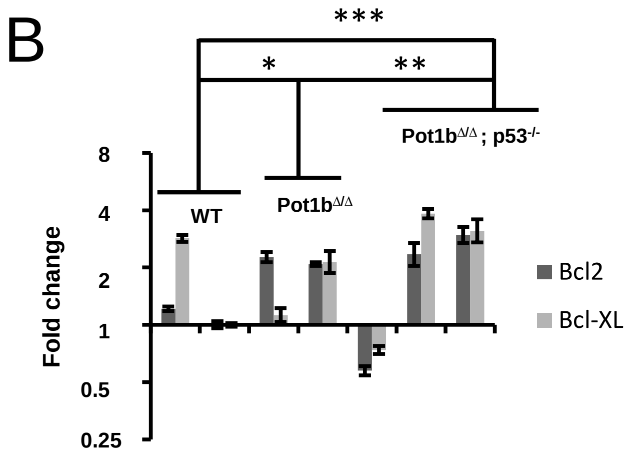

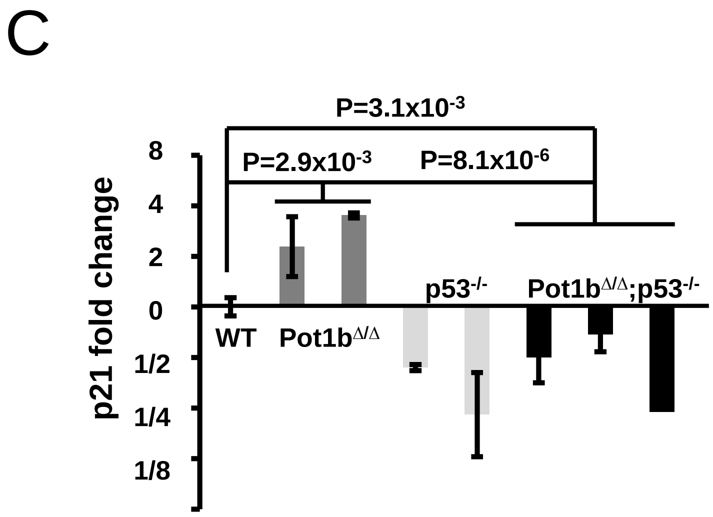

Supplementary Figure 5

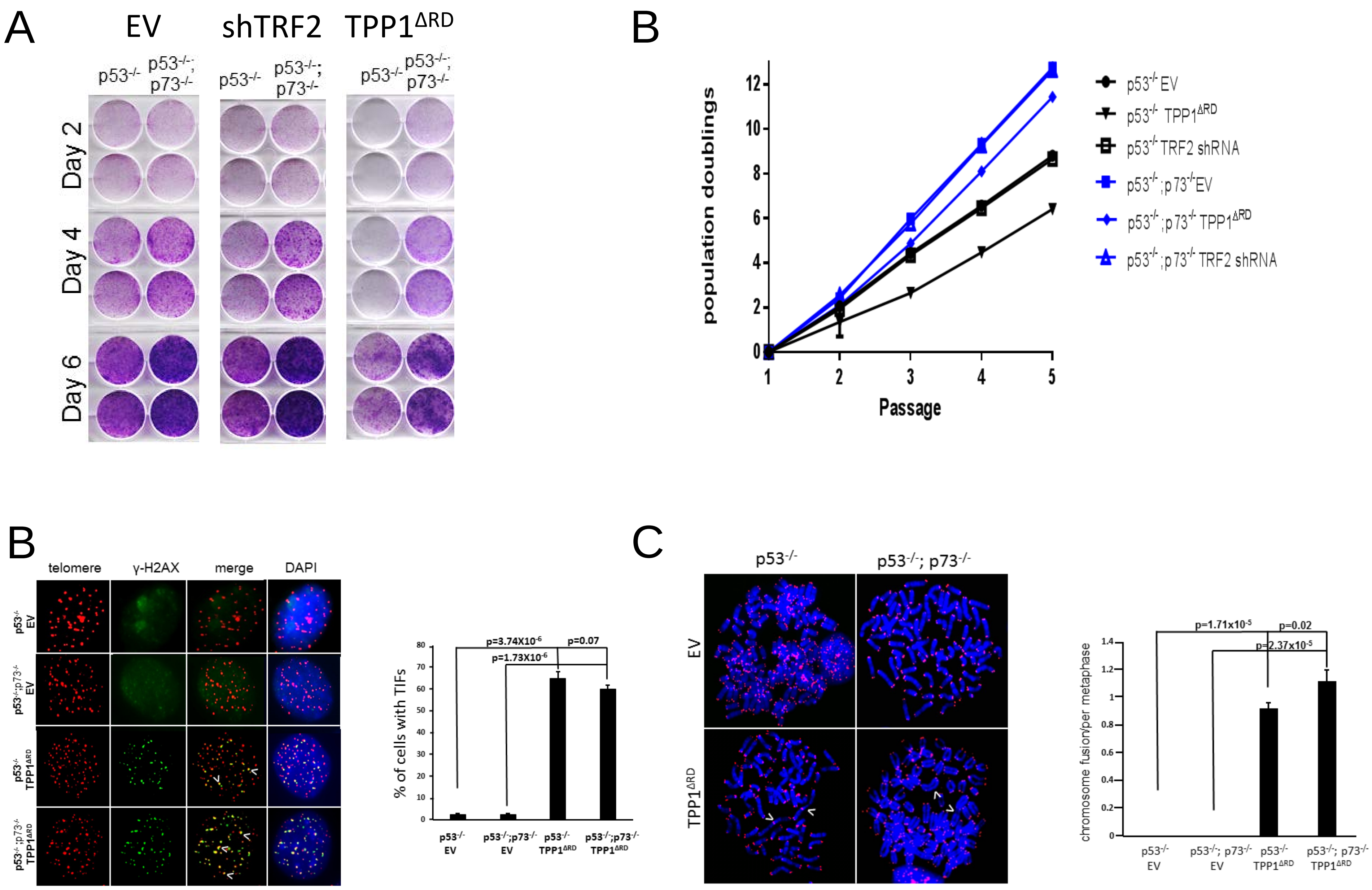

Supplementary Figure 6

Model

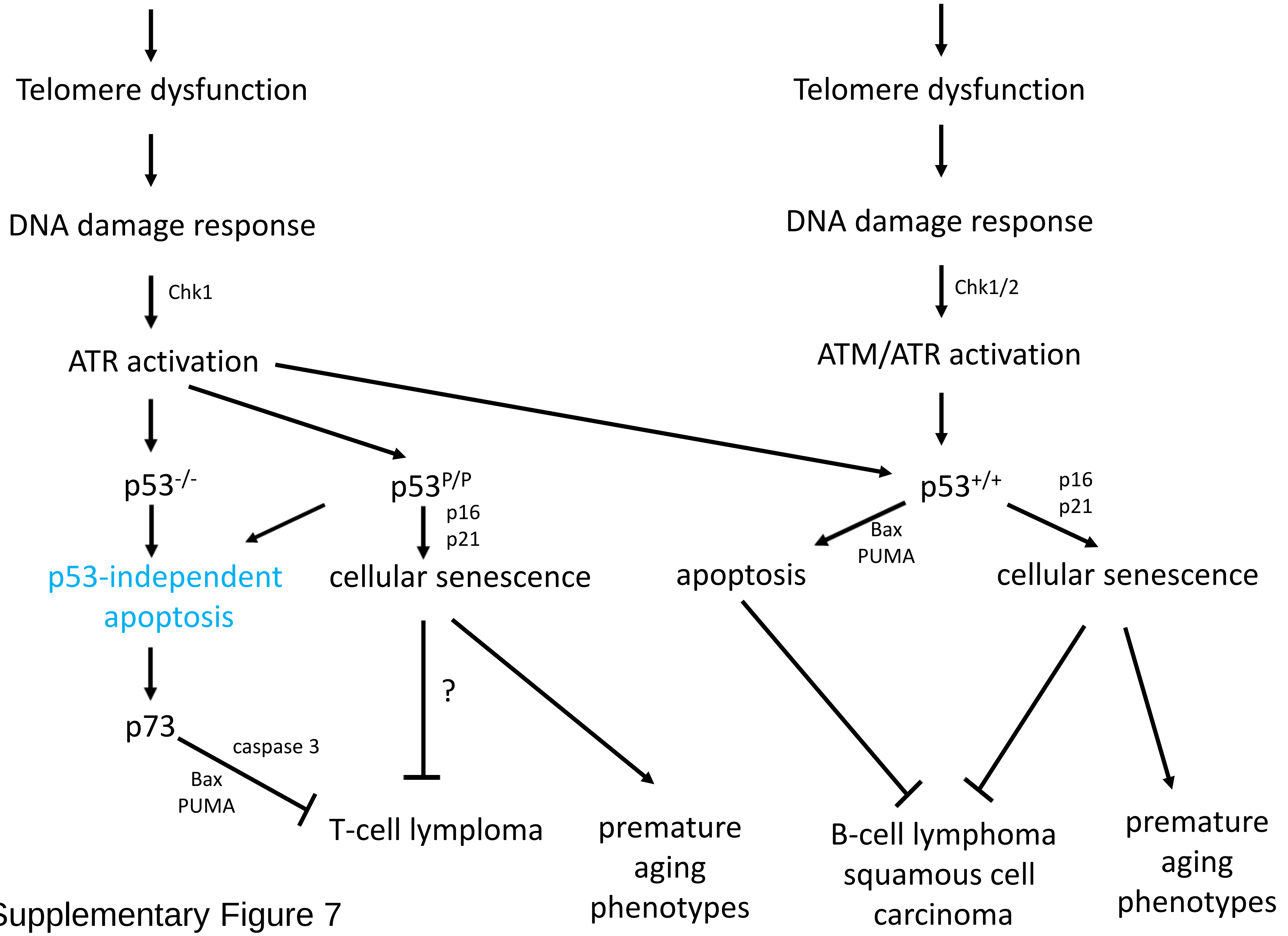

Supplementary Figure 7
